# Unculturable bacteria exploit a secretory protein to antagonize insect melanization for persistent infection

**DOI:** 10.1128/mbio.01896-25

**Published:** 2025-08-29

**Authors:** You Li, Yu Du, Dongsheng Ren, Yu Bin, Qian Chen, Taiyun Wei

**Affiliations:** 1 State Key Laboratory of Agricultural and Forestry Biosecurity, Fujian Agriculture and Forestry University 12449, Fuzhou, Fujian, China; The University of Mississippi Medical Center, Jackson, Mississippi, USA

**Keywords:** *Candidatus *Liberibacter asiaticus, huanglongbing, melanization, secretory protein, psyllid, persistent infection

## Abstract

Phloem-inhabiting unculturable bacterial pathogens are persistently transmitted by insect vectors. However, how they evade insect immune responses to ensure persistent transmission remains unknown. The important melanization immune response in insects is triggered by cleavage of prophenoloxidase (PPO) into active phenoloxidase (PO) via clip-domain serine proteases (CLIPs). Here, we demonstrate that infection of *Candidatus* Liberibacter asiaticus (*C*Las) in psyllid vectors activates the peptidoglycan recognition protein (PGRP)-CLIP1-CLIP4-PPO-PO signaling cascade to induce a mild melanization response, ensuring persistent *C*Las infection without causing significant insect fitness costs to the insect. A *C*Las-encoded secretory protein, SDE3230, directly interacts with PGRP and suppresses its activity in transducing this signaling cascade. CLIP4 cleaves PPO between arginine 125 and methionine 126 residues to active PO to induce melanization, and this cleavage pattern in psyllid is distinct from other insects. However, SDE3230 competitively binds to this cleavage site of PPO with CLIP4, thereby suppressing PPO effective cleavage. Collectively, these findings reveal the dual role of SDE3230 in facilitating the mild melanization response, benefitting persistent *C*Las infection and insect fitness.

**IMPORTANCE:**

Psyllid-borne huanglongbing is the most destructive citrus disease worldwide, causing billions of dollars in annual production losses and threatening the entire citrus industry. Currently, the mechanism by which the causal agent *Candidatus* Liberibacter asiaticus (*C*Las) antagonizes psyllid innate immune responses to facilitate its coexistence with psyllid vectors is still unknown. Here, we report that *C*Las exploits the highly expressed secretory protein SDE3230 in psyllids to suppress the important melanization immune response in hemolymph via inhibiting the pattern recognition receptor PGRP activity and the cleavage of prophenoloxidase into active phenoloxidase by clip-domain serine proteases. The pattern of PPO cleavage is novel, and this process ultimately ensures persistent *C*Las infection and insect fitness. Our findings provide insights into how *C*Las has evolved novel strategies to evade the insect melanization response, thereby facilitating persistent *C*Las transmission.

## INTRODUCTION

Phloem-inhabiting unculturable bacterial pathogens, most notably phytoplasmas, *Candidatus* (*Ca*.) Liberibacters, and *Ca*. Phlomobacters, are exclusively transmitted by piercing-sucking insects, such as psyllids and leafhoppers, and cause devastating disease outbreaks in citrus and other crop plants (1, 2). Asian citrus psyllid, *Diaphorina citri*-transmitted *Ca*. Liberibacter asiaticus (*C*Las), which causes citrus huanglongbing (HLB or citrus greening), is currently considered the most destructive citrus disease worldwide (3). It affects all commercial citrus cultivars and causes significant economic losses due to reduced yield, rendering fruit inedible, fruit drop, and even tree death (4). *C*Las is transmitted by *D. citri* in a persistent-propagative manner (5). *C*Las infects various *D. citri* organs, including salivary glands, filter chambers, fat bodies, guts, and reproductive organs (5–7). During its circulative infection in *D. citri*, *C*Las encounters multiple innate immune systems, including phagocytosis, melanization, coagulation, nodulation, and encapsulation (8). Developing a deeper understanding of the balanced interplay relationship for *C*Las transmission, insect fitness, and innate antibacterial immunity will help elucidate how phloem-inhabiting unculturable bacterial pathogens adapt and coexist with their vectors.

The melanization response is one of the most immediate and universal immune responses, specifically occurring in the hemolymph of insects (9, 10). Upon encountering invading pathogens, pattern recognition receptors (PRRs), such as peptidoglycan recognition proteins (PGRPs), sequentially activate downstream serine protease cascades, many of which possess a regulatory clip domain and are called clip-domain serine proteases (CLIPs) (11, 12). These enzymes are usually secreted into the extracellular space in their zymogen forms (proCLIPs) and are activated by cleavage at the amino end of the catalytic domain, enabling them to perform multiple functions (13). These CLIPs cleave prophenoloxidase (PPO), an inactive zymogen and the rate-limiting enzyme in melanogenesis, at a conserved peptide bond to proteolytically activate phenoloxidase (PO) (8). There are three mechanisms in the final step of PPO cleavage to produce activated PO in some species of insects (14–16). However, it is unknown how PPO cleavage and activity are enabled by serine proteases in other insects. The PPO activation cascade is negatively regulated by the serine protease inhibitor (serpin) superfamily in insects, such as *Aedes aegypti*, *Bombyx mori*, and *Helicoverpa armigera* (17). PO is the terminal protease in the serine protease cascade and catalyzes phenol oxidation to quinones (8). The spontaneous polymerization of quinones forms melanin, the black pigment, at the infection site, which encapsulates and neutralizes invaders (9, 17). Melanization functions in cuticle sclerotization, wound healing, and pathogen elimination (18). Melanization weakens the activity and infection of *Autographa californica* multicapsid nucleopolyhedrovirus (AcMNPV) in silkworm *B. mori*, serves as an antifungal mechanism in the greater wax moth *Galleria mellonella*, and defends against Bt toxin in the Asian corn borer *Ostrinia furnacalis* (19–21). This melanization is closely associated with insect survival during pathogen invasion (18). However, it remains unknown how the melanization response plays an antibacterial role during the persistent infection of *C*Las in insect vectors. Furthermore, it is still unknown how *C*Las antagonizes the melanization response to ensure persistent bacterial transmission by insect vectors.

Due to its undeciphered growth factors, *C*Las is unculturable, and its pathogenicity remains unclear. However, it is known that *C*Las has a complete Sec-dependent secretory machinery, enabling secretory proteins to traverse the cytoplasmic membrane and be released into host cells (22). Secretory proteins via the Sec-dependent pathway possess an N-terminal signal peptide, which facilitates the potential translocation of secretory proteins into host cells (23). It has been reported that 86 proteins of *C*Las are predicted to contain a signal peptide that directs them to be translocated outside the cytoplasm, potentially facilitating *C*Las-host plant interactions (22). Sec-delivered effector 1 (SDE1, CLIBASIA_05315) of *C*Las inhibits citrus papain-like cysteine proteases via interaction, thereby reducing citrus defense responses (24). SDE15 (CLIBASIA_04025) of *C*Las suppresses plant immunity and promotes *C*Las propagation by targeting the citrus-susceptible gene *ACCELERATED CELL DEATH 2* (25). SDE3 (CLIBASIA_00420) of *C*Las inhibits autophagy in citrus plants to promote *C*Las infection, thereby accelerating HLB in citrus (26). Secretory proteins also exhibit differential expression in plant hosts and insect vectors at the transcriptional level. For example, CLIBASIA_03230 (SDE3230) is highly expressed in the psyllid vector compared to the plant host for reasons that remain unknown (23). However, it is still unknown whether the secretory proteins of *C*Las are involved in regulating innate immune responses of *D. citri* to ensure persistent bacterial transmission by insect vectors. In this study, we reveal how the secretory protein SDE3230 of *C*Las effectively antagonizes *D. citri* melanization immune response to facilitate the coexistence of pathogens with their vectors.

## RESULTS

### Induced melanization response inhibits *C*Las infection and increases psyllid fitness

**Fig 1:**
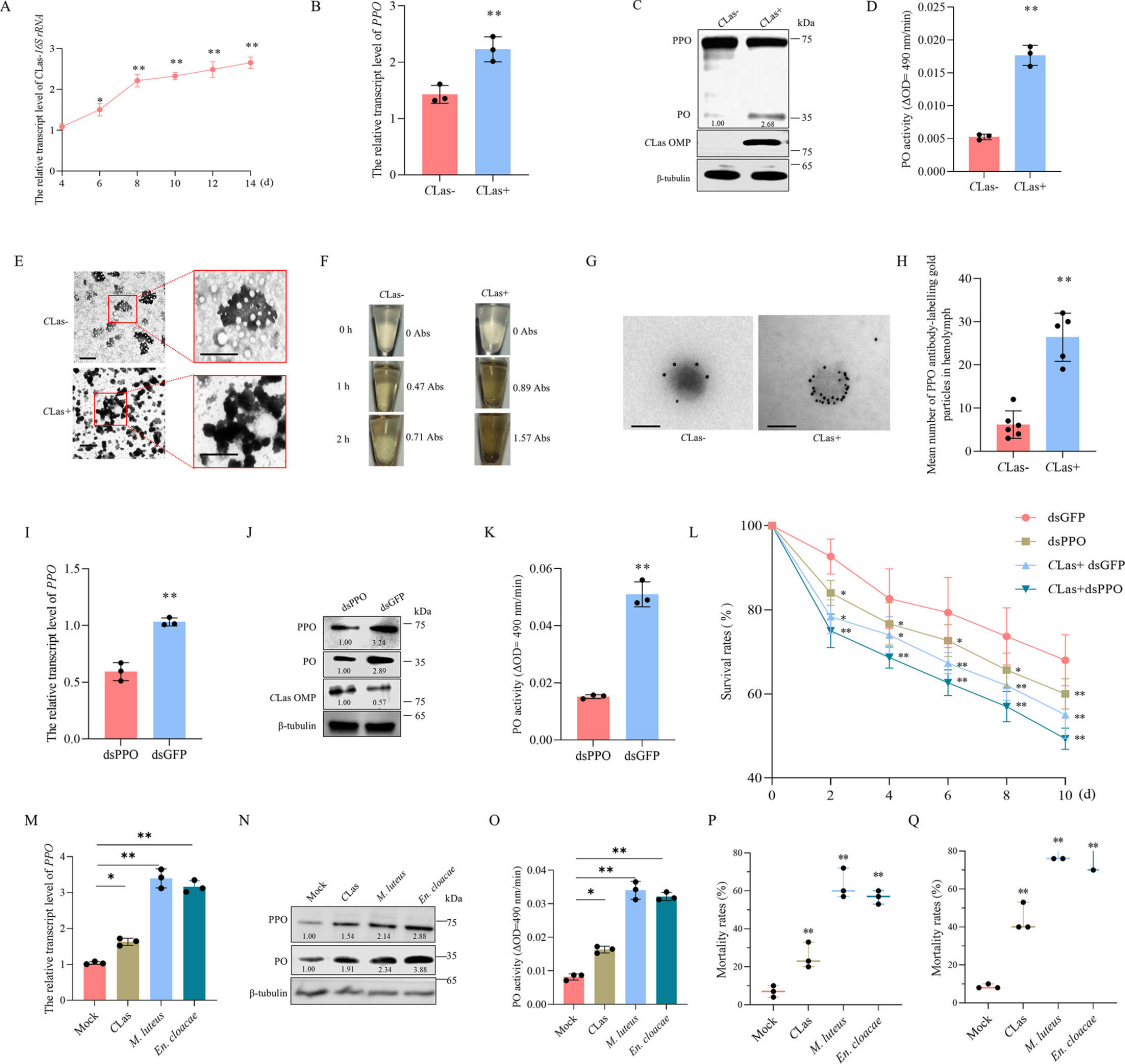
*C*Las infection induces an antibacterial melanization response in psyllid vector. (**A**) RT-qPCR assays measuring the relative transcript levels of *C*Las 16S rRNA at 4–14 days padp. (**B**) RT-qPCR assay determining the relative transcript levels of PPO in *C*Las-infected (at eight days padp) and uninfected psyllids. (**C**) Western blot assay detecting the cleavage of PPO into PO in the hemolymph of *C*Las-infected (at eight days padp) and uninfected psyllids. (**D**) PO activities in the hemolymph of *C*Las-infected (at eight days padp) and uninfected psyllids. (**E**) Transmission electron microscopy showing the melanin aggregates in the hemocytes of *C*Las-uninfected or infected (at eight days padp) psyllids. The boxed areas were enlarged to clearly display the melanin aggregates. Bars, 500 nm. (**F**) *In vitro* spontaneous melanization assay of hemocytes from *C*Las-uninfected and infected (at eight days padp) psyllids. Melanin production was recorded by photography at various time points from 0 to 2 h. (**G**) Negative staining-based immunoelectron microscopy showing the localization of PPO on melanin in the hemolymph of *C*Las-uninfected or infected (at eight days padp) psyllids. The hemolymph crude extracts were immunolabeled with PPO-specific IgG as the primary antibody, followed by treatment with 10 nm gold particle-conjugated IgG as the secondary antibody. Bars, 100 nm. (**H**) The mean number of PPO antibody-labeling gold particles in the hemolymph. Means (±SD) represent six replicates, each consisting of 30 hemolymph samples. (**I–K**) The relative transcript levels of *PPO* detected by RT-qPCR assay (**I**); protein accumulation levels of PPO, PO, and *C*Las OMP detected by Western blot assay (**J**); and PO activities (**K**) in dsGFP- or dsPPO-treated *C*Las-infected psyllids. (**L**) The survival rates of dsGFP- or dsPPO-treated *C*Las-uninfected or infected psyllids at different days post-microinjection. (**M–O**) The relative transcript levels of *PPO* detected by RT-qPCR assays (**M**), protein accumulation levels of PPO and PO (**N**), and PO activities (**O**) in psyllids at six days after injection with *C*Las, *M. luteus*, or *En. cloacae*. (**P**) The mean mortality rates of psyllids at six days after injection with *C*Las, *M. luteus*, or *En. cloacae*. (**Q**) The mean mortality rates of dsPPO-treated psyllids at six days after injection with *C*Las, *M. luteus*, or *En. cloacae*. Data in panels A, B, D, I, K, L, M, O, and P represent means (±SD) of three replicates, with each replicate containing 30 insects (two-tailed *t*-test). *P < 0.05; **P < 0.01. The * and ** in panel L were specific to the comparison to dsGFP in each day post microinjection. Relative protein levels in panels C, J, and N are quantified using ImageJ with β-tubulin as a reference protein. The data represent three replicates, with each replicate containing 30 insects.

First, we investigated whether melanization response defends against *C*Las infection in psyllid vectors. It was found that the transcript levels of *C*Las 16S rRNA increased rapidly from four to eight days post-first access to diseased plants (padp) and then remained relatively stable (Fig. 1A). This suggests that the anti-*C*Las immune systems of psyllids are notably activated at eight days padp. *C*Las infection significantly increased the transcript levels of PPO, the key melanization protein, at eight days padp (Fig. 1B). Furthermore, *C*Las infection promoted the cleavage of PPO into a 35 kDa protein, consistent with the predicted molecular weight of PO at eight days padp (Fig. 1C). Accordingly, PO activities in *C*Las-infected psyllids were higher than in *C*Las-uninfected controls at eight days padp (Fig. 1D). This suggests that the melanization response specifically occurs in the insect hemolymph. It was found the higher accumulation levels of PPO and PO in the hemolymph compared to other tissues (Fig. S1A and B). Electron microscopy showed that more melanin aggregates were formed in the hemolymph of *C*Las-infected psyllids compared to *C*Las-uninfected controls (Fig. 1E), consistent with *in vitro* spontaneous melanization assays (Fig. 1F). Negative staining-based immunoelectron microscopy revealed dense distribution of PPO on the hemocytes from *C*Las-infected psyllids, whereas PPO was sparsely distributed on the hemocytes from *C*Las-uninfected control (Fig. 1G and H). Taken together, these results suggest that *C*Las infection in psyllids induces active melanization in the hemolymph.

To understand the effect of melanization response on *C*Las infection, *C*Las-infected or uninfected psyllids were microinjected with *in vitro* synthesized dsRNAs targeting PPO or GFP (dsPPO or dsGFP). Knocking down *PPO* expression in *C*Las-infected psyllids significantly reduced the accumulation and activity of PO but increased the accumulation of *C*Las (Fig. 1I through K; Fig. S2A). Thus, the activated melanization response possesses anti-*C*Las activity. To address the biological significance of melanization response for psyllids, the survival rates of *C*Las-infected or uninfected psyllids after the knockdown of PPO expression were examined. It was found that the survival rate of dsGFP-treated *C*Las-infected psyllids was significantly lower than that of dsGFP-treated *C*Las-uninfected controls (Fig. 1L), suggesting that *C*Las potentially poses a disadvantage for psyllid survival. Furthermore, the survival rate of dsPPO-treated *C*Las-infected psyllids was notably lower than that of dsGFP-treated *C*Las-infected controls (Fig. 1L). Thus, the reduced melanization response via dsPPO treatment significantly increases *C*Las accumulation and insect mortality rates. We also microinjected *C*Las, along with *Micrococcus luteus* and *Enterobacter cloacae*, two known pathogenic bacteria for insects, into psyllids (27, 28). At six days post-injection, *M. luteus* and *En. cloacae* infection significantly increased PPO transcript levels, PPO and PO protein accumulation levels, and PO activity levels compared to *C*Las infection (Fig. 1M through O; Fig. S2B). Moreover, dsPPO-treated psyllids infected with *M. luteus* and *En. cloacae* showed a significantly higher mortality rate than *C*Las-infected psyllids (Fig. 1P and Q). These results suggest that *C*Las evolved lower virulence than *M. luteus* and *En. cloacae* to dsPPO-treated psyllids and reflect its adaptation to insects to a certain extent. The immune response in psyllids during *C*Las propagation effectively controls the excessive *C*Las accumulation, thereby potentially regulating psyllid fitness and facilitating the coexistence of *C*Las with its vectors.

### *C*Las infection activates CLIP4 expression for directly cleaving PPO into active PO

**Fig 2:**
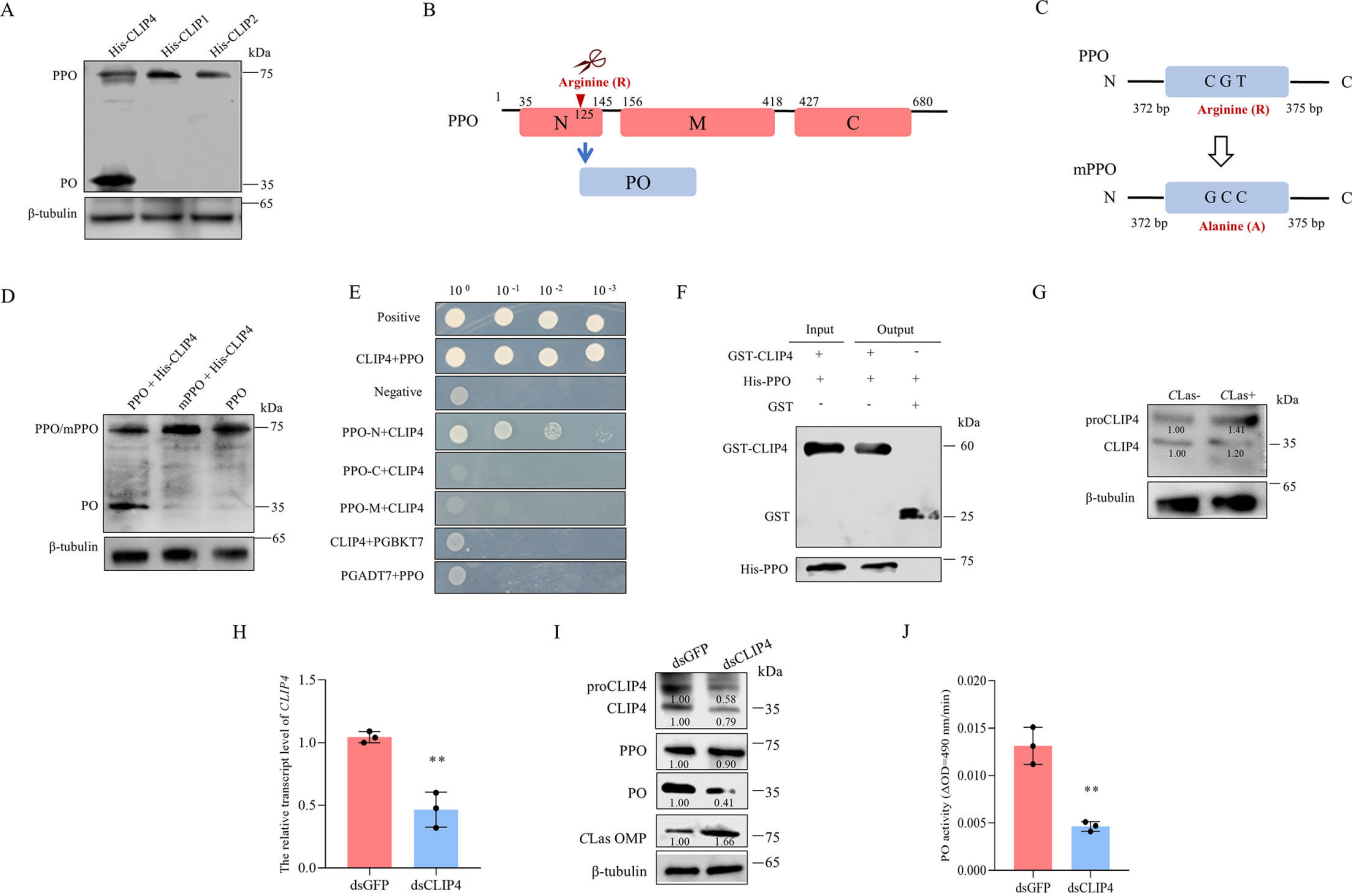
*C*Las infection activates the expression of *CLIP4*, facilitating the direct cleavage of PPO into PO. (**A**) Detection of CLIPs’ cleavage ability after incubating purified His-CLIP1, His-CLIP2, or His-CLIP4 with purified PPO *in vitro*, as determined by Western blot assays. (**B**) Schematic illustration of PPO cleavage into the PO subunit at the cleavage site between arginine 125 and methionine 126. (**C**) Schematic illustration of mutated PPO (mPPO), where arginine at position 125 of PPO is substituted with alanine. (**D**) Detection of PPO cleavage sites after incubating purified His-CLIP4 with purified PPO or mPPO *in vitro*, as determined by Western blot assays. (**E**) Y2H assays for detecting interactions between CLIP4 and different segments of PPO. Positive control: pGBKT7-53/pGADT7-T. Negative control: pGBKT7-Lam/pGADT7-T. Yeast cultures were grown in SD/-Trp-Leu-His-Ade medium. (**F**) GST pull-down assay for detecting the interaction of CLIP4 with PPO. (**G**) Western blot assay showing the cleavage of proCLIP4 into CLIP4 in *C*Las-uninfected or infected (at eight days padp) psyllids. (**H–J**) The relative transcript levels of *CLIP4* detected by RT-qPCR assays (**H**); protein accumulation levels of ProCLIP4, CLIP4, PPO, PO, and *C*Las OMP detected by Western blot assays (**I**); and PO activities (**J**) in dsGFP- or dsCLIP4-treated *C*Las-infected psyllids at eight days padp. Data in panels H and I represent means (±SD) of three replicates, with each replicate containing 30 insects (two-tailed *t*-test). **P < 0.01. The relative protein levels in panels G and I were quantified using ImageJ with β-tubulin as a reference protein. The data represents three replicates, with each replicate containing 30 insects.

Given that PPO is cleaved into PO by serine proteases, many of which are CLIPs (12, 29), we aimed to identify which CLIPs are involved in *C*Las-activated mild melanization response in psyllids. CLIP1 to CLIP5 homologs of *D. citri* were identified from GenBank (XP_017303373, XP_008488274, XP_026687172, XP_026678648, and XP_008488275) (Fig. S3A and Tables S1 and S2). RT-qPCR assays showed that *C*Las infection significantly increased the transcript levels of CLIP1, CLIP2, and CLIP4 in psyllid*s* (Fig. S3B), suggesting that *C*Las infection activates their expression. Next, the ability of CLIPs to cleave PPO was examined. The purified CLIP1, CLIP2, and CLIP4 were individually incubated with purified PPO *in vitro*. Western blot assays showed that the incubation of CLIP4, but not CLIP1 or CLIP2, led to the cleavage of PPO, thereby generating PO (Fig. 2A). This suggests that CLIP4 serves as the serine protease responsible for directly cleaving PPO into PO.

Liquid chromatography coupled with tandem mass spectrometry (LC-MS/MS) analysis showed that one potential cleavage site of PPO to generate the 35 kDa PO was localized at the N-terminus of PPO, specifically at residue 125 (arginine [R]) (Fig. 2B; Fig. S3C). The arginine at position 125 was mutated into alanine (A), resulting in mPPO (Fig. 2C). The purified mPPO or PPO was respectively incubated with CLIP4, and Western blot assays showed that mPPO was not cleaved by CLIP4 (Fig. 2D). Yeast two-hybrid (Y2H) and GST pull-down assays demonstrated that CLIP4 specifically interacted with the N-terminal segment of PPO containing the cleavage site (35–145 aa), but did not interact with the other segments of PPO (Fig. 2E and F). Thus, CLIP4 specifically recognizes and binds to PPO, initiating the cleavage of PPO into PO between the arginine and methionine residue at position 125-126.

It is well known that insect *C*LIP4 is generated from the cleavage of proCLIP4, the zymogen form of CLIP4 (30). Western blot assays showed the higher accumulation levels of proCLIP4 and cleaved CLIP4 in *C*Las-infected psyllids (Fig. 2G; Fig. S2C). Knocking down CLIP4 expression decreased the cleavage of proCLIP4 into CLIP4, which consequently led to reduced cleavage of PPO into PO, inhibited PO activity, and promoted *C*Las accumulation (Fig. 2H through J; Fig. S2D). Thus, CLIP4 may be the key serine protease that directly cleaves PPO into PO, thereby inducing downstream melanization processes.

### *C*Las infection activates the PGRP-CLIP1-CLIP4-PPO-PO signaling cascade to induce melanization response

**Fig 3:**
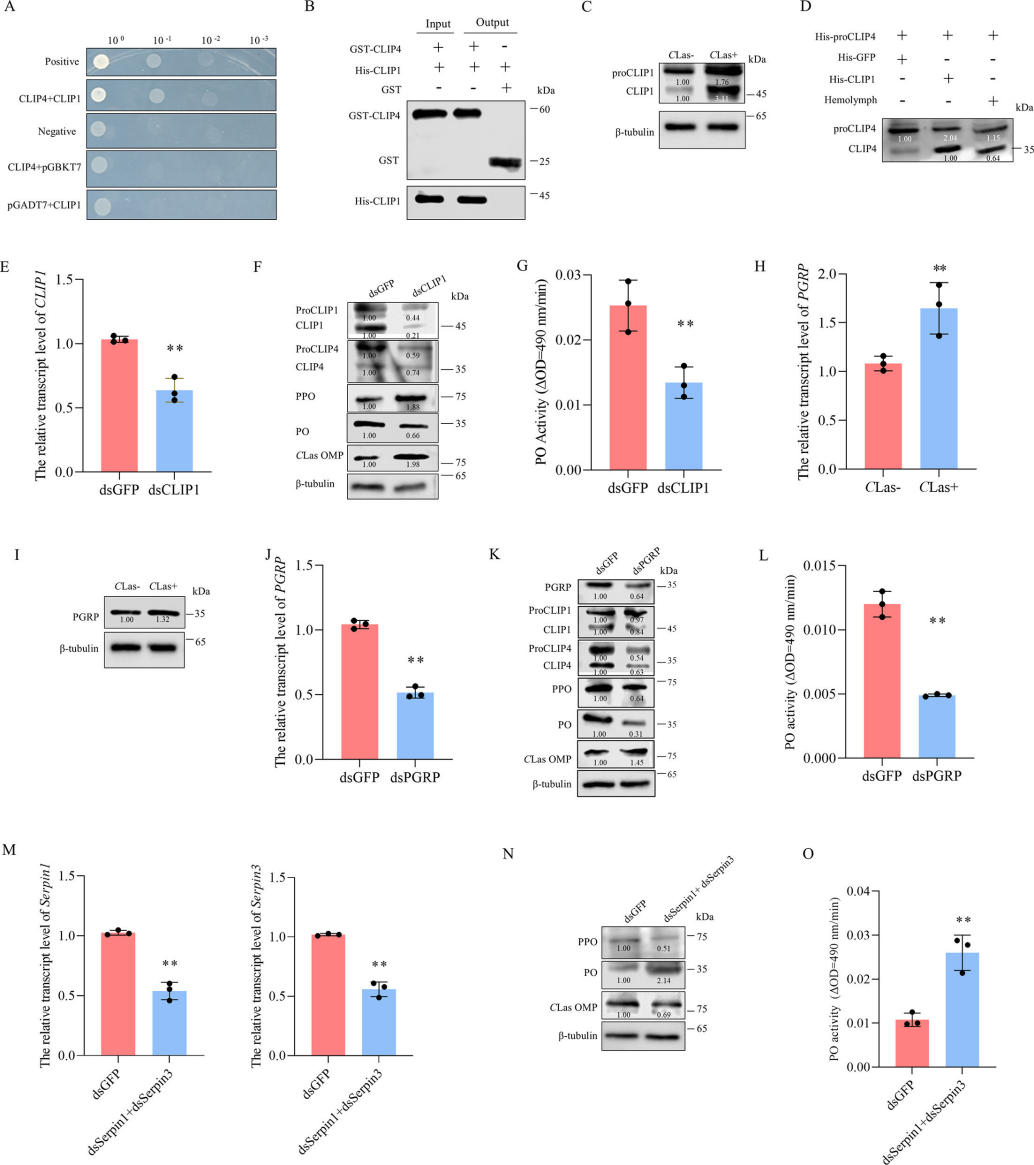
*C*Las infection activates the PGRP-CLIP1-CLIP4-PPO-PO signaling cascade in psyllid vector. (**A**) Y2H assay for detecting the interaction of CLIP4 with CLIP1. Positive control: pGBKT7-53/pGADT7-T. Negative control: pGBKT7-Lam/pGADT7-T. Yeast cultures were grown in SD/-Trp-Leu-His-Ade medium. (**B**) GST pull-down assay for detecting the interaction of CLIP4 with CLIP1. (**C**) Western blot assay showing the cleavage of proCLIP1 into CLIP1 in *C*Las-uninfected or infected (at 8 days padp) psyllids. (**D**) Detection of CLIP1 cleavage ability after incubating purified His-CLIP1 with purified His-proCLIP4 *in vitro*, as determined by Western blot assays. The hemolymph crude extracts served as the control. (**E–G**) The relative transcript levels of *CLIP1* detected by RT-qPCR assays (**E**); the protein accumulation levels of ProCLIP1, CLIP1, ProCLIP4, CLIP4, PPO, PO, and *C*Las OMP detected by Western blot assays (**F**); and PO activities (**G**) in dsGFP- or dsCLIP1-treated *C*Las-infected psyllids at 8 days padp. (**H, I**) The relative transcript and protein accumulation levels of PGRP in *C*Las-uninfected or infected (at eight days padp) psyllids. (**J–L**) The relative transcript levels of *PGRP* detected by RT-qPCR assays (**J**); the protein accumulation levels of PGRP, ProCLIP1, CLIP1, ProCLIP4, CLIP4, PPO, PO, and *C*Las OMP detected by Western blot assays (**K**); and PO activities (**L**) in *C*Las-infected (at eight days padp) psyllids. (**M–O**) The relative transcript levels of *Serpin1* or *Serpin3* detected by RT-qPCR assays (**M**); the protein accumulation levels of PPO, PO, and *C*Las OMP detected by Western blot assays (**N**); and PO activities (**O**) in dsGFP- or dsSerpin1+dsSerpin3-treated *C*Las-infected (at eight days padp) psyllids. Data in panels E, G, H, J, L, M, and O represent means (±SD) of three replicates, with each replicate containing 30 insects (two-tailed *t*-test). **P < 0.01. Relative protein levels in panels C, D, F, I, K, and N were quantified using ImageJ with β-tubulin as a reference protein. The data represents three replicates, with each replicate containing 30 insects.

Given that the conversion of PPO into PO is driven by a cascade of serine proteases (29), we next examined the relationship between CLIP4 and other CLIPs. Y2H and GST pull-down assays showed an interaction between CLIP4 and CLIP1 (Fig. 3A and B). Western blot assays revealed that *C*Las infection promoted the accumulation of proCLIP1, the zymogen of CLIP1, which was subsequently cleaved to generate CLIP1 (Fig. 3C). *In vitro* cleavage assays showed that the purified CLIP1 effectively cleaved proCLIP4 into CLIP4 (Fig. 3D). Knocking down CLIP1 expression in *C*Las-infected psyllids significantly reduced the cleavage of proCLIP4 into CLIP4, resulting in decreased cleavage of PPO into PO, which ultimately inhibited PO activity and promoted *C*Las infection (Fig. 3E through G; Fig. S2E). Thus, CLIP1 serves as the serine protease that directly interacts with and mediates the cleavage of proCLIP4 into CLIP4. Altogether, the results reveal that the CLIP1-CLIP4-PPO-PO signaling cascade is responsible for the CLas-induced melanization response in psyllids.

In general, the initiation of melanization response involves the recognition of pathogens by pattern recognition receptors (PRR), including peptidoglycan recognition proteins (PGRPs) (31). Unexpectedly, only one PGRP (GenBank accession XM_026822519, Tables S1 and S2) without transmembrane helices and signal peptide was identified in the *D. citri* genome (Fig. S4). RT-qPCR and Western blot assays showed that *C*Las infection elevated both the transcript and protein levels of PGRP (Fig. 3H and I). Knockdown of PGRP expression significantly decreased the cleavage of proCLIP1, proCLIP4, and PPO into their respective subunits, ultimately inhibiting PO activity and promoting *C*Las accumulation (Fig. 3J through L; Fig. S2F). These results demonstrate that *C*Las infection triggers PGRP to transduce the CLIP1-CLIP4-PPO-PO signaling cascade, thereby inducing a downstream melanization response.

The serpin superfamily of *D. citri*, comprising six homologs (Serpin1 to Serpin6; GenBank accessions XP_008487511, XP_026678781, XP_026686116, XP_026677956, XP_008472813, and XP_017300323), acts as negative regulators of the PPO activation cascade (Fig. S5A). *C*Las infection upregulated the transcript levels of *Serpin1*, *Serpin3*, *Serpin4*, and *Serpin6* (Fig. S5B and C). Notably, *Serpin1* and *Serpin3* exhibited the highest expression levels (Fig. S5B). The simultaneous knockdown of *Serpin1* and *Serpin3* expression notably increased the cleavage of PPO into PO, ultimately enhancing PO activity and suppressing *C*Las accumulation (Fig. 3M through O; Fig. S2G). Therefore, it is evident that serpins are activated by *C*Las infection to mediate the mild melanization response.

### Secretory protein SDE3230 of *C*Las competitively binds to PPO, thereby inhibiting the cleavage of PPO by CLIP4

**Fig 4:**
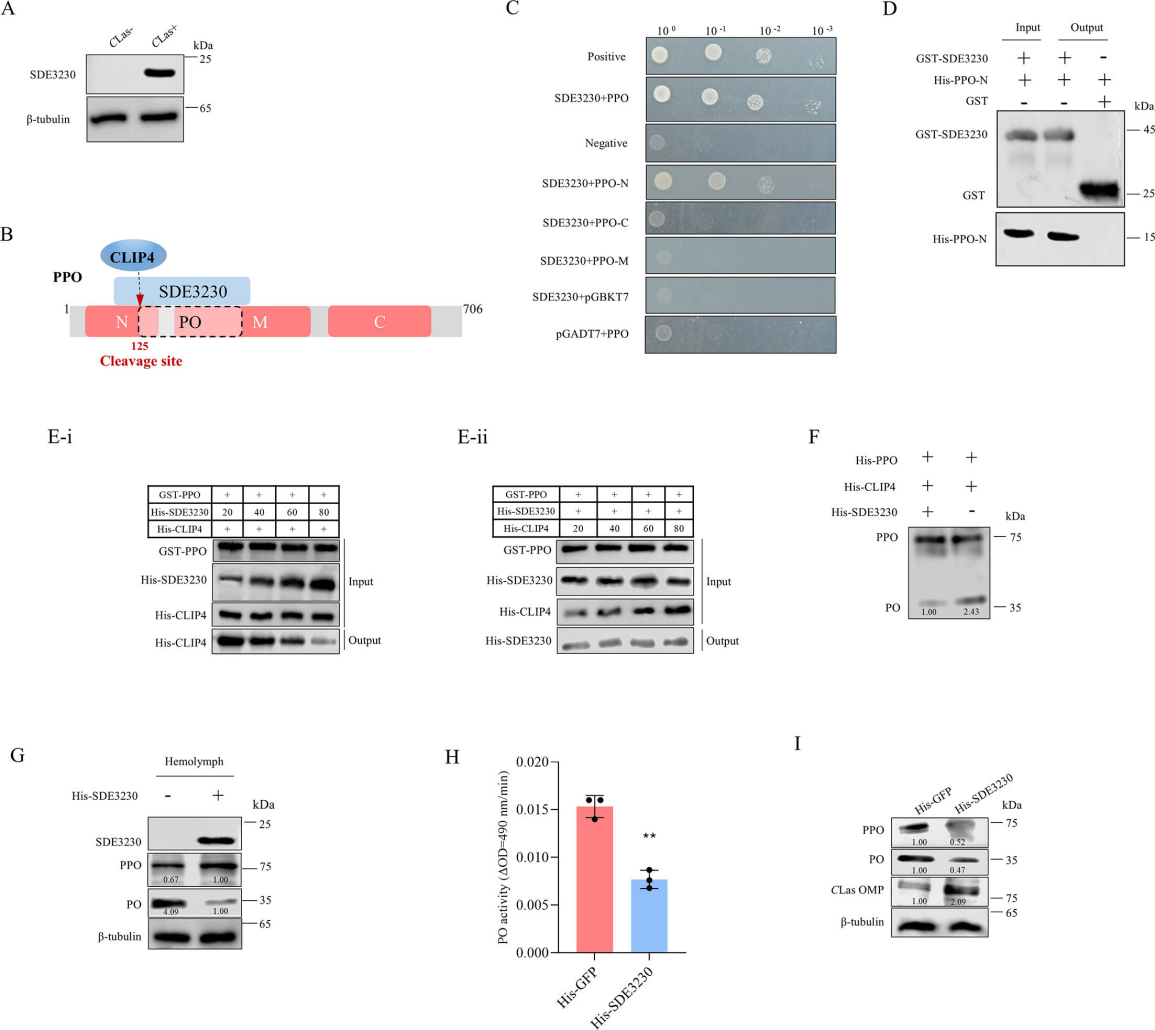
SDE3230 competitively binds to PPO to inhibit the cleavage of PPO by *C*LIP4. (**A**) Western blot assays for detecting the accumulation of SDE3230 in *C*Las-uninfected or infected (at eight days padp) psyllids. (**B**) Schematic illustration of SDE3230 preventing CLIP4 from cleaving PPO. (**C**) Y2H assay for detecting the interaction of SDE3230 with PPO segments. Positive control: pGBKT7-53/pGADT7-T. Negative control: pGBKT7-Lam/pGADT7-T. Yeast cultures were grown in SD/-Trp-Leu-His-Ade medium. (**D**) GST pull-down assay showing the interaction of SDE3230 with PPO-N. SDE3230 fused with GST served as the bait, GST as the control, and PPO-N fused with His as the prey. (**E**) GST pull-down assays revealed competitive interactions among PPO, SDE3230, and CLIP4. GST-PPO and His-CLIP4 were initially incubated with glutathione-Sepharose beads, followed by the addition of His-SDE3230 to the beads. The amounts of His-SDE3230 increased (20, 40, 60, or 80 µL) with a decrease in the binding between PPO and CLIP4, as shown in panel E-i. GST-PPO and His-SDE3230 were incubated with glutathione-Sepharose beads, and then, His-CLIP4 was added to the beads. When the amounts of His-CLIP4 were increased (20, 40, 60, or 80 µL), the binding between PPO and SDE3230 was not affected, as shown in panel E-ii. The relative intensities of bands are shown below. (**F**) Detection of SDE3230 inhibiting the cleavage of PPO by CLIP4 after incubating purified His-SDE3230, His-CLIP4, or His-PPO *in vitro*, as determined by Western blot assays. (**G**) Detection of SDE3230 inhibiting the cleavage of PPO after incubating purified His-SDE3230 with the hemolymph crude extracts *in vitro*, as determined by Western blot assays. (**H**) Detection of SDE3230 inhibiting PO activity. (**I**) Detection of the accumulation of PPO, PO, and *C*Las OMP in *C*Las-infected (at eight days padp) psyllids after microinjection of purified His-SDE3230 or His-GFP, as determined by Western blot assays. Data in panel H represent means (±SD) of three replicates, with each replicate containing 30 insects (two-tailed *t*-test). **P < 0.01. Relative protein levels in panels F, G, and I were quantified using ImageJ with β-tubulin as a reference protein. The data represents three replicates, with each containing 30 insects.

We investigated the mechanism through which *C*Las induces mild melanization response in psyllids. A previous study demonstrated that among 86 tested secretory proteins, SDE3230 of *C*Las exhibited the highest expression level (22). Western blot assay confirmed the specific expression of SDE3230 in *C*Las-infected psyllids (Fig. 4A). Subsequently, we used SDE3230 as a bait protein to screen for its interactors in the melanization response. Y2H and GST pull-down assays showed the interaction of SDE3230 with the N-terminal segment (35–145 aa) of PPO containing the cleavage site (Fig. 4B through D). Given that CLIP4 also interacts with the same N-terminal segment of PPO, we hypothesized that SDE3230 competes with CLIP4 for binding to PPO, thereby suppressing PPO cleavage. Competitive GST pull-down assays were performed to compare the binding affinities for SDE3230-PPO and CLIP4-PPO. GST-PPO and His-CLIP4 were initially incubated with glutathione-Sepharose beads, followed by the addition of His-SDE3230. The results indicated that increased concentrations of His-SDE3230 led to a decrease in the binding level of CLIP4-PPO (Fig. 4E–i). Meanwhile, GST-PPO and His-SDE3230 were incubated with glutathione-Sepharose beads, followed by the addition of varying amounts of His-CLIP4 to the beads. Interestingly, the binding between PPO and SDE3230 was not affected regardless of the quantity of His-CLIP4 present (Fig. 4E–ii). These results indicate that SDE3230 competes with CLIP4 for binding to PPO *in vitro*.

We then investigated whether SDE3230 could effectively inhibit CLIP4-mediated PPO cleavage. The purified His-CLIP4 and His-PPO were co-incubated with purified His-SDE3230 *in vitro*, resulting in reduced generation of PO cleaved from PPO (Fig. 4F). Next, we examined the ability of SDE3230 to suppress PPO cleavage in psyllid hemolymph by incubating purified SDE3230 with samples of the extracted psyllid hemolymph. SDE3230 effectively reduced the cleavage of PPO into PO in hemolymph, leading to reduced PO activity (Fig. 4G and H). Similarly, microinjection of purified SDE3230 into *C*Las-infected psyllids reduced the cleavage of PPO into PO while increasing *C*Las accumulation (Fig. 4I). Thus, it is evident that SDE3230 inhibits the cleavage of PPO into PO, thereby suppressing melanization response and facilitating *C*Las infection.

### SDE3230 suppresses PGRP activity to inhibit the initiation of melanization response

**Fig 5:**
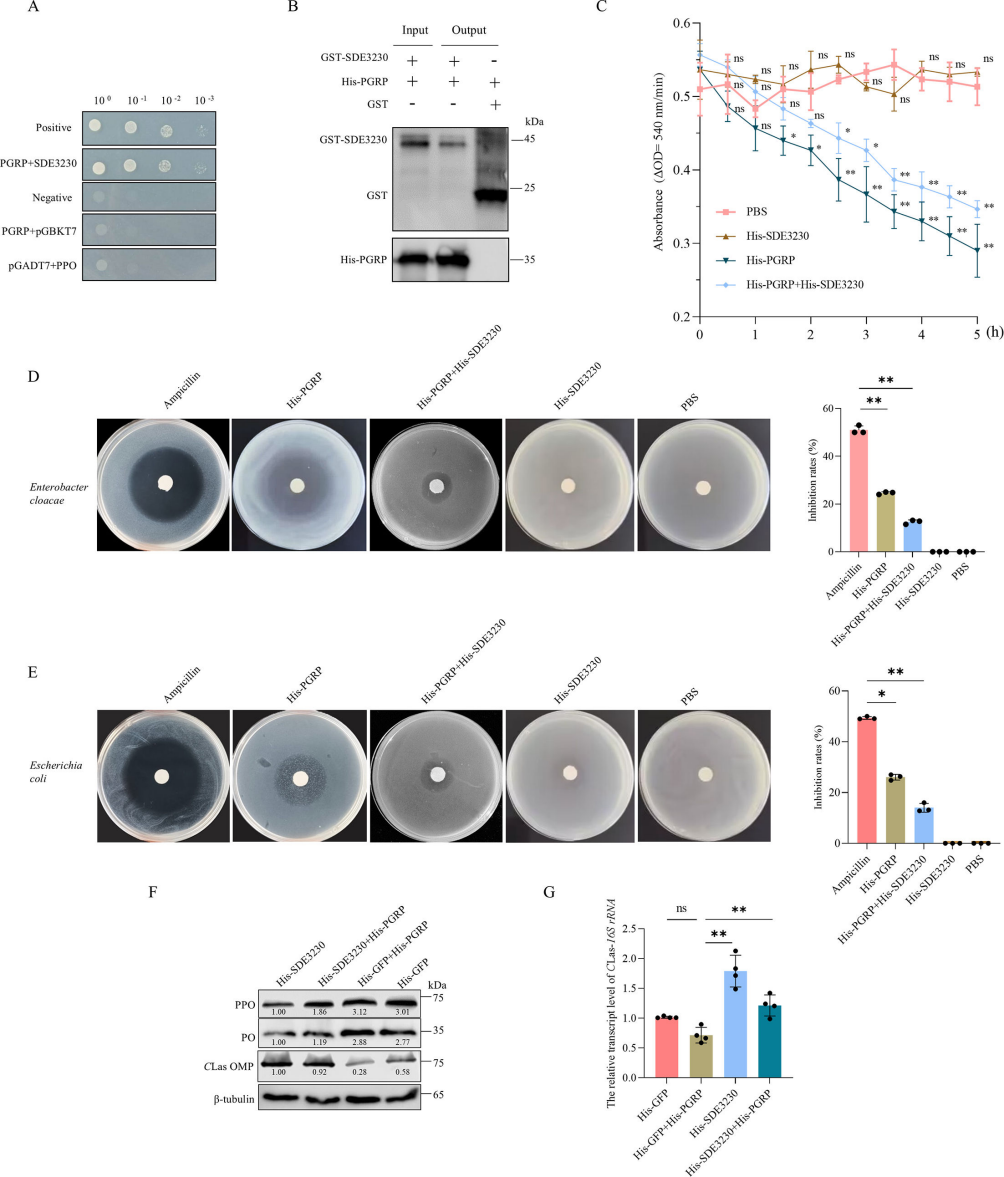
SDE3230 interacts with and suppresses PGRP activity in transducing the downstream melanization response. (**A**) Y2H assay for detecting the interaction of PGRP with SDE3230. Positive control: pGBKT7-53/pGADT7-T. Negative control: pGBKT7-Lam/pGADT7-T. Yeast cultures were grown in SD/-Trp-Leu-His-Ade medium. (**B**) GST pull-down assay for detecting the interaction of PGRP with SDE3230. (**C**) Kinetics of *Es. coli* peptidoglycan degradation. Enzymatic activity was recorded at 540 nm every 30 min for 5 h. Amidase activity assay showing the effects of SDE3230 inhibiting PGRP amidase activity. PBS served as the negative control. Data represent means (±SD) of three replicates. ns, not significant; *P < 0.05; **P < 0.01. (**D, E**) Zones of inhibition assays showing the effects of SDE3230 suppressing PGRP activity in the antibacterial effects against two Gram-negative bacillus strains. The plates were inoculated with *En. cloacae* (**D**) and *Es. coli* (**E**). Paper disks impregnated with His-PGRP alone, a mixture of His-PGRP and His-SDE3230, and His-SDE3230. Ampicillin served as a positive control, with PBS as a negative control. The diameters of inhibition zones were measured to calculate the inhibition rates. Data represent means (±SD) of three replicates. *P < 0.05; **P < 0.01. (**F, G**) Effects of the microinjection of purified His-SDE3230 or His-PGRP on the protein accumulation levels of PPO, PO, and *C*Las OMP (**F**) or the transcript levels of *C*Las *16S rRNA* (**G**) in *C*Las-infected (at eight days padp) psyllids, as detected by Western blot assay and RT-qPCR assays, respectively. Relative protein levels in panel F were quantified using ImageJ with β-tubulin as a reference protein. The data represents three replicates, with each containing 30 insects. Data in panel G represents means (±SD) of three replicates, with each containing 30 insects (two-tailed *t*-test). ns, not significant; *P < 0.05; **P < 0.01. The ns, *, and ** in panel C indicate the statistical significance relative to the PBS control for each day post-microinjection.

*C*Las infection triggered PGRP to transduce the CLIP1-CLIP4-PPO-PO signaling cascade, thereby inducing a downstream melanization response (Fig. 3). Y2H and GST pull-down assays demonstrated the interaction of PGRP with SDE3230 (Fig. 5A and B). Some insect PGRPs function as an amidase, specifically degrading peptidoglycan from Gram-negative bacteria (32, 33). We then investigated whether psyllid PGRP also possesses amidase activity and examined whether the interaction of PGRP with SDE3230 affects PGRP activity. After incubating PGRP with SDE3230, the absorbance of *Escherichia coli* peptidoglycan was higher compared to PGRP alone (Fig. 5C), indicating that psyllid PGRP can degrade peptidoglycan from Gram-negative bacteria, and this amidase activity was inhibited by SDE3230. Furthermore, bacterial inhibition assays proved that PGRP exhibited antibacterial effects on *En. cloacae* and *Es. coli*, while the combination of SDE3230 with PGRP significantly decreased the antibacterial effect of PGRP (Fig. 5D and E). Thus, SDE3230 potentially suppresses PGRP activity. Moreover, co-injection of SDE3230 with PGRP suppressed PGRP-mediated increase in PO activity in psyllids (Fig. 5F). Accordingly, PGRP injection decreased *C*Las accumulation, whereas co-injection of PGRP and SDE3230 increased *C*Las accumulation (Fig. 5F and G). These results indicate that SDE3230 can inhibit the initiation of melanization response by interacting with PGRP. Overall, these results show that PGRP recognizes *C*Las infection, thus triggering downstream melanization response. To achieve persistent infection, *C*Las exploits SDE3230 to suppress PGRP accumulation, thereby inhibiting downstream melanization response.

## DISCUSSION

Invading pathogens usually activate the insect immune response, resulting in fluctuations in the expression of immune genes, tissue damage, or cytopathology to defend against pathogen infection (9). The melanization response in insects plays a vital role in defending against pathogen invasion. For example, the melanization response in *Galleria mellonella* demonstrates broad-spectrum resistance to various fungi by directly eliminating invading fungi (21). AcMNPV infection in the hemolymph of *Ostrinia furnacalis* activates the antiviral melanization response (34). In contrast, insect-borne pathogens that establish persistent infection neither cause high insect mortality nor are completely eradicated by the immune system, indicating a balanced immune interaction between pathogens and insects (35). This balance represents a defense mechanism evolved by insect vectors that allow pathogens to establish persistent infection, ensuring the survival of the insects (36). For example, rice stripe virus and rice stripe mosaic virus inhibit the cleavage ability of PPO in the hemolymph of planthopper or leafhopper vectors, thereby attenuating hemolymph melanization response and facilitating viral persistent propagation (37, 38). Phloem-inhabiting unculturable bacterial pathogens cause devastating crop disease outbreaks that include Huanglongbing in citrus caused by *D. citri*-transmitted *C*Las. Here, we reveal how *C*Las exploits its highly expressed secretory proteins to antagonize the melanization immune response, facilitating its coexistence with the transmitted vectors. To the best of our knowledge, our model provides the first mechanistic framework to elucidate how phloem-inhabiting unculturable bacterial pathogens modulate insect vector immune responses, enabling efficient transmission through insect vectors.

The long-term association between insect-transmitted pathogens and their vectors results in evolutionary trade-offs that balance insect fitness costs with persistent pathogen transmission (35). *D. citri* and *C*Las infection reach a metastable equilibrium that defines the state of persistent infection. This persistent transmission induces immune homeostasis to regulate the coexistence of *D. citri* and *C*Las. Here, we discover that *C*Las infection activates the PGRP-CLIP1-CLIP4-PPO-PO signaling cascade, inducing a mild anti-*C*Las melanization response without apparent fitness costs to the insect. In contrast, *M. luteus* and *En. cloacae* in *D. citri* induce a robust antibacterial melanization response, resulting in high insect mortality rates. Interestingly, further suppression of melanization response by knocking down PPO expression in *C*Las-infected psyllids leads to increased *C*Las accumulation and higher insectmortality. Therefore, the controlled suppression of melanization response during *C*Las infection effectively manages excessive *C*Las accumulation, thereby facilitating the coexistence of *C*Las with its vectors. These findings have broad implications for understanding the establishment of persistent infection by vector-borne bacterial pathogens in insect vectors in natural environments.

We further reveal that *C*Las secretory protein SDE3230 effectively antagonizes the vector melanization response. Unlike most Gram-negative bacteria, *C*Las possesses only a type I secretion system and Sec-dependent secretory machinery, with at least 86 secretory proteins identified (22). *C*Las secretory proteins have been shown to suppress immunity and promote *C*Las infection in plant hosts (24, 39). For instance, SDE4405 promotes *C*Las infection via suppressing host autophagy through interaction with ATG8, while SDE3 directly associates with citrus cytosolic glyceraldehyde-3-phosphate dehydrogenases to inhibit plant immunity (26). Among all tested secretory proteins, SDE3230 exhibits the highest expression level in *D. citri*, indicating its potential critical role in suppressing the vector immune response (22). *D. citri* CLIP4 directly recognizes the cleavage site, arginine at position 125 of PPO, and cleaves PPO into the active PO to induce the melanization response. However, SDE3230 also recognizes the cleavage site of PPO. Furthermore, SDE3230 specifically interacts with PPO rather than CLIP4, and its binding ability to PPO is stronger than that of CLIP4. Therefore, SDE3230 effectively inhibits the ability of CLIP4 to directly cleave PPO into PO, thereby suppressing the melanization response. Noteworthy, the pattern of cleaving PPO is distinct from known three mechanisms of insect PPO activation (29). This cleavage is similar to the *D. melanogaster* recombinant PPO1 (GenBank accession: AAF57775) with cleavage sites in both N-terminal and C-terminal (40).

**Fig 6:**
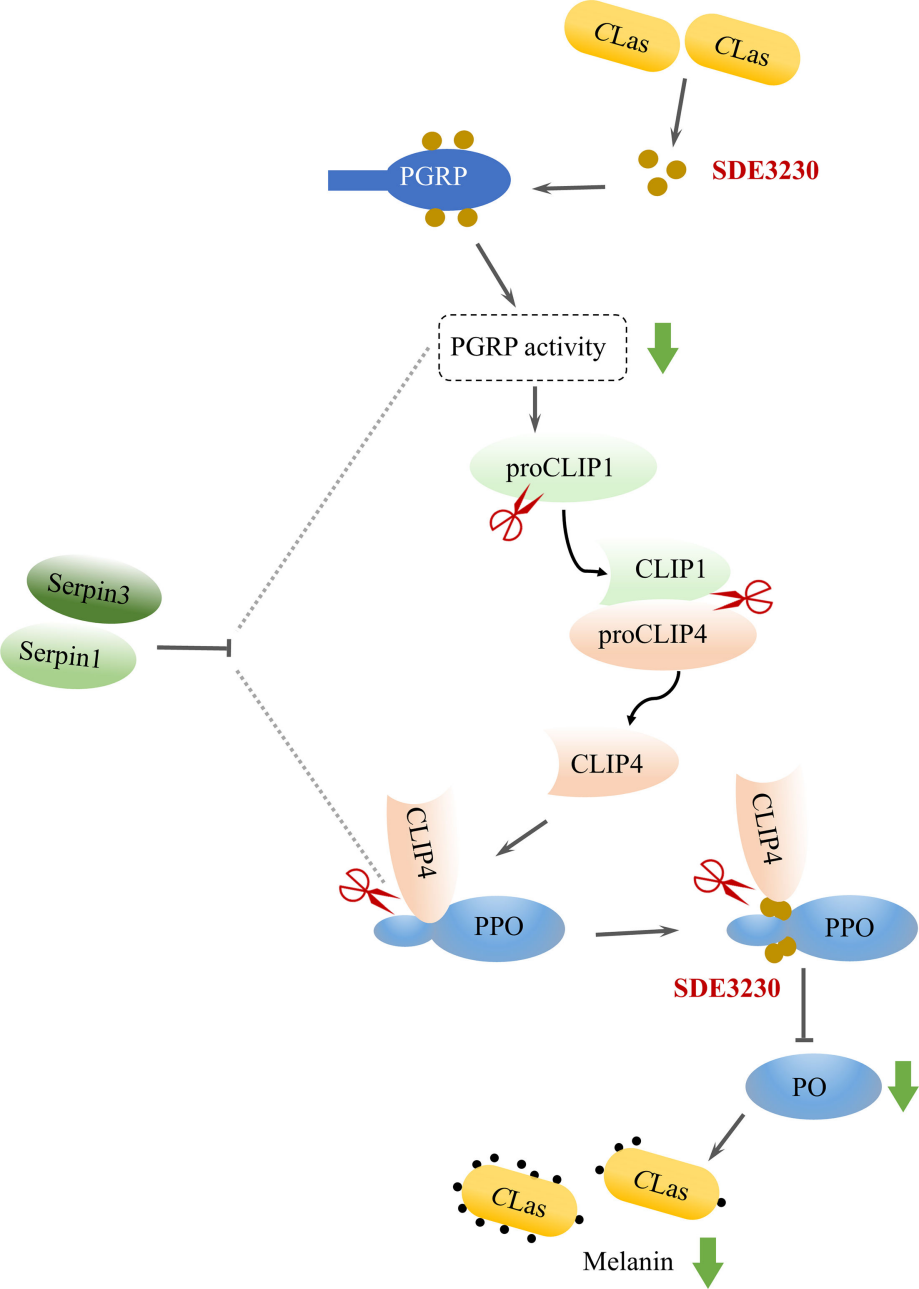
Proposed model of *C*Las infection triggers a mild melanization response in psyllid vectors. *C*Las infection is recognized by PGRP, which then activates downstream serine protease cascades. ProCLIP1 is cleaved into CLIP1, which subsequently cleaves proCLIP4 into CLIP4. CLIP4, in turn, cleaves PPO into PO, initiating anti-*C*Las melanization response. Meanwhile, Serpin1 and Serpin3 are activated to control the melanization response. *C*Las exploits SDE3230 to suppress the melanization response via inhibiting PGRP activity or the cleavage of PPO by CLIP4 through competitively binding to PPO. This balance between mild melanization response and *C*Las facilitates persistent infection.

In invertebrates, the melanization pathway primarily relies on PRRs, which recognize pathogen-associated molecular patterns (PAMPs) to initiate downstream effectors against pathogen infections (41). Consequently, the recognition of *C*Las PAMPs by PGRP activates the downstream intracellular CLIP1-CLIP4-PPO-PO signaling cascade to induce a mild antibacterial melanization response. Unexpectedly, only one PGRP was found in the *D. citri* genome. This PGRP may functionally resemble *Drosophila* PGRP-LB, which could reduce immunostimulatory peptidoglycan levels. Interaction between this PGRP and the *C*Las secretory protein SDE3230 effectively decreases PGPR amidase activity, ultimately inhibiting the downstream melanization response triggered by PGRP (42). In summary, we propose a model wherein *C*Las antagonizes vector melanization immune signaling to facilitate persistent bacterial infection (Fig. 6). When *C*Las infects the psyllid, it is recognized by PGRP, triggering activation of the serine proteinase cascade, including the activation and cleavage of proCLIP1 into CLIP1, subsequently leading to the cleavage of proCLIP4 into CLIP4. Subsequently, the cleaved CLIP4 serves as the serine proteinase, directly cleaving PPO into active PO to induce the melanization response. Meanwhile, the highly expressed *C*Las-secreted protein SDE3230 in *D. citri* plays a dual role. It inhibits the cleavage of PPO into active PO by CLIP4 and suppresses the PGRP-activated CLIP1-CLIP4-PPO-PO signaling cascade, thereby inducing a mild antibacterial melanization response. Thus, *C*Las has evolved to exploit the secretory protein SDE3230 to evade the melanization response, thereby facilitating its coexistence with *D. citri*. This delicate balance among bacterial transmission, insect fitness, and innate immunity tolerance potentially contributes to the global spread of this destructive pest and its associated phytopathogen.

## MATERIALS AND METHODS

### Insects, antibodies, and *C*Las

*C*Las-uninfected psyllid *D. citri* individuals were originally collected from orange jessamine (*Murraya paniculata*) plants at Fujian Agriculture and Forestry University in Fuzhou City, Fujian Province, China. These psyllids were reared on orange jessamine plants and propagated for several generations under greenhouse conditions of 25 ± 1°C, 60% ± 5% relative humidity, and a 16-h light/8-h dark cycle. The offspring of each generation were tested using PCR assays to ensure the absence of *C*Las and maintain the *C*Las-uninfected colony. The *C. sinensis* var. *brasiliensis* Tanaka plants exhibiting typical HLB symptoms were collected from Gutian, Fujian Province, China, and confirmed to be *C*Las-infected using PCR assays.

Polyclonal antibodies against PPO, outer membrane protein (OMP) of *C*Las, CLIP4, CLIP1, PGRP, and SDE3230 were prepared as described previously (43). IgG was isolated from the antisera using a protein A-Sepharose affinity column (Thermo Fisher Scientific, USA) and then eluted in PBS. Mouse monoclonal antibodies against GST, His, and β-tubulin were purchased from TransGene Biotech (China).

### Phylogenetic analyses

Phylogenetic analyses of serpins, CLIPs, and PGRP amino acid sequences were conducted using MAFFT (https://mafft.cbrc.jp/alignment/software) under default parameters. Ambiguously aligned regions were trimmed using Geneious 9.1.5. Maximum likelihood analyses were performed using RAxML2.0 with 1,000 bootstrap replications (44). The resulting tree was visualized and edited in iTOL (https://itol.embl.de).

### Effect of *C*Las infection on expression of melanization-related proteins

To compare the mRNA expression levels of PPO in different tissues of psyllids, we dissected the heads, alimentary canals, and hemolymph. Next, total RNAs were extracted and subjected to RT-qPCR assays using the 2× RealStar Fast SYBR qPCR Mix (High ROX; Genstar, A303) on the QuantStudio 5 Real-Time PCR System (Thermo Fisher Scientific) to determine the relative mRNA expression levels of PPO. The transcript levels of the housekeeping gene elongation factor 1 alpha (*EF1α*) served as the internal reference for normalizing the gene expression levels. Relative gene expression levels were calculated using the ^−ΔΔ^CT (cycle threshold) method (45).

The protein expression levels of PPO and its cleaved PO subunit in different psyllid tissues were analyzed using Western blot assays. The total proteins from the heads, alimentary canals, and hemolymph of psyllids were extracted using RIPA lysis buffer (Thermo Fisher Scientific, 89901). The primary antibodies against PPO and β-tubulin (0.5 µg/µL) were used, while goat anti-rabbit IgG-peroxidase (Sigma-Aldrich, A0545) at a concentration of 0.5 µg/µL served as the secondary antibody. The experiments were replicated at least three times, with each replicate using a pool of 30 psyllids. Bands corresponding to β-tubulin were used to verify protein loading. Band intensities of proteins analyzed by Western blot assays were quantified using ImageJ software (Table S3).

To investigate the expression pattern of melanization-related proteins during *C*Las infection in psyllids, over 400 *C*Las-uninfected psyllids were allowed to feed on *C*Las-infected *C. sinensis* var. *brasiliensis* Tanaka plants. At 4, 6, 8, 10, 12, and 14 days padp, psyllids were individually examined for the presence of 16S rRNA of *C*Las using RT-PCR assays. Subsequently, *C*Las-positive psyllids were individually tested for the expression of PPO, 16S rRNA, serpins, CLIPs, and PGRP using RT-qPCR assays. In addition, the accumulation of PPO, PO, CLIPs, *C*Las OMP, and PGRP was detected using Western blot assays.

### LC-MS/MS analyses of PPO cleavage

The 35 kDa PO proteins from the hemolymph of *C*Las-infected psyllids were isolated via SDS-PAGE gel and then analyzed using mass spectrometry at APTBIO (Shanghai, China). The PO peptide was identified with Mascot (https://www.matrixscience.com/) and assembled using Geneious 9.0.2.

### PO activity assays

To investigate PO activity, hemolymph crude extracts derived from 30 psyllids were collected. In brief, the abdomen of psyllids was dissected with a pin on slide glass and immersed with 10 µL of pre-cooled PBS. The hemolymph crude extracts from each test were examined using Fluorometer Spectrophotometer DS11 (DeNovix, USA) to confirm the same protein concentrations. Then, the solutions were collected and incubated with 50 µL of 0.5 mM dopamine in 10 mM Tris-HCl buffer (pH 8.0) at room temperature for 5 min. The samples were measured for absorbance at 490 nm every 5 min, using Synergy H1 Hybrid Reader (BioTek, USA). The slopes of absorbance change were used to determine PO activity. Three biological replicates were utilized for each experiment.

### Electron microscopy

To examine melanization response in the hemolymph, at eight days padp, psyllids were dissected to collect the hemolymph, which was then diluted in sterile H_2_O. The samples were then subjected to phosphotungstic acid (PTA) negative staining. Briefly, hemolymph crude extracts were dropped onto a paraffin film and covered with a carbon-free aromatic film copper mesh for 10–15 min. Next, the copper mesh was successively incubated with 10 µL of 2% PTA for 3 min and finally baked under a baking lamp for 3 min. The resulting samples were examined using a transmission electron microscope (Hitachi, H-7650).

To immunolabel PPO in the hemolymph, a nickel mesh was blocked with a blocking buffer for 2 min, followed by incubation with hemolymph crude extracts for 15 min. After washing three times, the nickel mesh was incubated with the primary antibody against PPO for 1.5 h, followed by incubation with goat anti-rabbit IgG conjugated with 10-nm-diameter gold particles (0.5 µg/µl; Abcam) as the secondary antibody for 1 h (Abcam). Finally, the samples were incubated with PTA for 5 min and examined using a transmission electron microscope.

### Effect of knocking down melanization-related protein expression on the melanization response, *C*Las infection, and psyllid survival

The T7 RNA polymerase promoter sequence 5′-ATTCTCTAGAAGCTTAATACGACTCACTATAGGG-3′ was added to the forward and reverse primers for amplifying the 500 bp or fragments without signal peptide of serpin1, serpin3, CLIP1, CLIP4, PGRP, PPO, or GFP at the 5′ terminal. The dsRNAs targeting each segment were synthesized *in vitro* using the T7 RiboMAX Express RNAi System (Promega Biotech, cat. P1700). At eight days padp, *C*Las-uninfected or infected psyllids were microinjected with dsRNAs (approximately 200 ng/leafhopper) using a Nanoject II Auto-Nanoliter Injector (Spring) and then transferred to orange jessamine plants for recovery. At four days post-injection, RT-qPCR and Western blot assays were performed to determine the accumulation levels of 16S rRNA of *C*Las, as well as the melanization-related proteins, using the methods described above. Meanwhile, PO activity in each treatment group was also investigated using methods described earlier. The survival rates of *C*Las-uninfected or infected psyllids after microinjection of dsRNAs targeting PPO (dsPPO) or GFP (dsGFP) were recorded at 2, 4, 6, 8, and 10 days post-injection.

### *In vitro* spontaneous melanization assay

At eight days padp, hemolymph derived from CLas-infected psyllids was diluted in 10 µL PBS. These hemolymph crude extracts were then incubated with 50 µL of 0.5 mM dopamine in PBS and allowed to spontaneously oxidize at room temperature. The melanization of the samples was photographed, and absorbance was measured at 490 nm at 0, 1, and 2 h post-incubation.

### CLIP4 cleaving PPO into PO *in vitro* and *in vivo*

The fragments without signal peptide of PPO, CLIP1, CLIP2, CLIP4, and GFP were cloned into the pET-28b vector to construct plasmids expressing His-tagged fusion proteins (His-PPO, His-CLIP1, His-CLIP2, His-CLIP4, and His-GFP). The recombinant proteins were individually expressed in the *Es. coli* strain BL21 and purified using High Affinity Ni-Charged Resin FF (Genscript, cat. L00666-25). Besides, the full-length ORFs of CLIP1, CLIP2, CLIP4, and GFP were cloned into the pFastBac1 vector, incorporating C-terminal His/Flag/Myc tags. Recombinant bacmids were generated in *Es. coli* DH10Bac (Thermo Fisher, 10361012) and subsequently transfected into *Spodoptera frugiperda* cell (sf9 cell) using Cellfectin II Reagent (Thermo Fisher, 10362100). Viral stocks were amplified through two passages, and protein expression was confirmed via Western blot assays. The concentration of recombinant proteins was tested by bicinchoninic acid assay. Next, 100 µL of purified PPO was incubated with 30 µL sf9 cell-expressed His-CLIP1, His-CLIP2, or His-CLIP4 at 30°C for 4 h. The reaction mixture was then analyzed by Western blot assays using antibody against the His-tag or PPO.

The fragment without signal peptide of mPPO, a PPO mutant where the arginine at position 125 was mutated to alanine, was cloned into the pET-28b vector to construct plasmids expressing His-tagged fusion proteins, generating His-mPPO. The recombinant His-mPPO protein was expressed in *Es. coli* strain BL21 and purified. Next, 100 µL of purified mPPO or PPO was incubated with 30 µL sf9 cell-expressed His-CLIP4 at 30°C for 4 h. The reaction mixture was analyzed by Western blot assays using antibody against PPO.

The purified His-CLIP4 or His-GFP proteins were dialyzed in PBS and then microinjected into *C*Las-infected psyllids at eight days padp. At two days post-injection, these psyllids were tested to determine the accumulation of CLIP4, proCLIP4, PPO, PO, and OMP in Western blot assays, as well as PO activity.

### CLIP1 cleaving proCLIP4 into CLIP4 *in vitro* and *in vivo*

Briefly, 100 µL of purified His-proCLIP4 were incubated with 30 µL of purified His-GFP, His-CLIP1, or hemolymph crude extracts from *C*Las-infected psyllids at 30°C for 4 h. The reaction mixture was analyzed by Western blot assays using CLIP4-specific antibody. Meanwhile, the purified sf9 cell expressed His-CLIP1 and His-GFP was dialyzed in PBS and then microinjected into *C*Las-infected psyllids at eight days padp. At two days post-injection, these psyllids were tested for the accumulation of CLIP4, proCLIP4, CLIP1, proCLIP1, PPO, PO, or OMP using Western blot assays.

### Yeast two-hybrid assays

The fragments without signal peptide of PPO and CLIP1, as well as the fragments of PPO-N, PPO-C, and PPO-M, were cloned into the pGBKT7 vector to create the bait plasmids, which were then transformed into the yeast strain AH109 to test for self-activation. On the other hand, the fragments without signal peptide of CLIP4, SDE3230, and PGRP were cloned into the pGADT7 vector to create the prey plasmids. Next, the bait and prey plasmids were co-transformed into the yeast strain AH109, followed by the selection of positive clones on double dropout (DDO) and quadruple dropout (QDO) medium plates. The interaction between pGBKT7-53 and pGADT7-T served as the positive control, whereas the interaction between pGBKT7-Lam and pGADT7-T was used as the negative control.

### GST pull-down assay

The fragments without signal peptide of CLIP4 and SDE3230 were cloned into the pGEX-4T-3 vector to construct plasmids expressing GST fusion protein as baits (GST-CLIP4 and GST-SDE3230). The fragments of PPO and PGRP without signal peptide, as well as the fragment of PPO-N (Fig. 2B) and CLIP1 (trypsin-like serine protease domain), were cloned into the pET-28b vector to construct plasmids expressing His fusion protein as preys (His-PPO, His-CLIP1, His-PGRP, and His-PPO-N). The recombinant proteins fused with the GST tag, along with GST alone, were separately expressed in *Es. coli* strain BL21. The lysates were then incubated with glutathione Sepharose 4B beads (Cytiva, cat. 17075605), followed by incubation with the recombinant proteins fused with the His-tag. Finally, the eluates were analyzed by Western blot assay using antibody against GST-tag or His-tag.

### Competitive binding assays

The competitive binding assay was performed to detect the varying binding affinities among SDE3230, CLIP4, and PPO. Briefly, the GST-PPO was incubated with glutathione Sepharose 4B beads at 4°C overnight. On the next day, the beads were washed with PBS and incubated with His-CLIP4, followed by incubation with 20, 40, 60, or 80 µL of His-SDE3230 for 5 h at 4°C. Following centrifugation and washing, the bead-bound proteins were separated by SDS-PAGE and analyzed using Western blot assays with antibody against GST-tag or His-tag.

GST-PPO was incubated with glutathione Sepharose 4B beads at 4°C overnight. The beads were then washed with PBS and incubated with His-SDE3230, followed by incubation with 20, 40, 60, or 80 µL of His-CLIP4 for 5 h at 4°C. Following centrifugation and washing, the bead-bound proteins were separated by SDS-PAGE and analyzed using Western blot assays with antibodies against GST or His.

### Effect of purified SDE3230 on cleavage of PPO in hemolymph and *C*Las infection

Hemolymph crude extracts derived from 100 *C*Las-uninfected psyllids were diluted in 200 µL PBS. The purified His-SDE3230 was then incubated with the hemolymph crude extracts. The incubation was performed for 4 h at 30°C. Subsequently, the accumulation of PPO or PO was analyzed using western blot assays.

The purified His-SDE3230 and GFP were dialyzed in PBS and then microinjected into *C*Las-infected psyllids at eight days padp. At two days post-injection, these psyllids were tested for the expression of *C*Las *16S rRNA* using RT-qPCR and the accumulation of PPO, PO, OMP, or PGRP using Western blot assays. Additionally, the PO activity was evaluated as described above.

### Effect of PGRP on *C*Las infection and PPO cleavage

The purified SDE3230, PGRP, or GFP was microinjected into *C*Las-infected psyllids at eight days padp. At one day post-injection, these psyllids were tested for the expression of *C*Las *16S rRNA* using RT-qPCR and the accumulation of PPO, PO, or OMP using Western blot assays.

### PGRP inhibiting bacterial assay

Equal volumes of pure cultures of *En. cloacae* and *Es. coli* solutions were added to sterile LB liquid medium before solidification. The mixture was then poured into 9 cm petri dishes and allowed to solidify. The round filter paper (diameter = 8 mm) was sterilized and saturated with ampicillin, His-SDE3230, His-PGRP, a mixture of His-SDE3230 and His-PGRP, and PBS. Next, the round papers were placed at the center of the media. The treatment with ampicillin served as the positive control, whereas the treatment with PBS was the negative control. The plates were incubated at 30°C for 72 h, after which the inhibition zones were measured.

### PGRP amidase activity assay

Insoluble peptidoglycan of *Es. coli* (1 mg/mL) was incubated with PBS, His-SDE3230, His-PGRP, and a mixture of His-SDE3230 with His-PGRP. Enzymatic digestion of peptidoglycan was tested as previously described with slight modifications (42). Enzymatic activity was measured by recording the optical clearance of the solution at 540 nm every 30 min for 5 h at 25°C using a Synergy H1 Hybrid Reader (BioTek, USA) with occasional shaking. Notably, a low absorbance indicates high degradation of peptidoglycan, specifically indicating high amidase activity.

### Effect of *M. luteus* or *En. cloacae* infection on PO accumulation and mortality of *D. citri*

*M. luteus* or *En. cloacae* was cultured at 30°C on potato dextrose agar (PDA) or Luria-Bertani (LB) medium plates. These cultures were diluted in PBS to an OD600 of 0.5 and then microinjected into psyllids. At 6 h post-injection, these psyllids were tested for the expression of *PPO* using RT-qPCR and the accumulation of PPO and PO using Western blot assays. The concentration of *M. luteus* and *En. cloacae* dilution referred to the median effect concentration (EC50) on *D. citri* (Fig. S6). In addition, the PO activity was evaluated as described above. The mortality of psyllids was also recorded. Notably, the motility rates of *M. luteus*, *En. cloacae*, or *C*Las-treated psyllids were recorded on different days post-injection.

### Statistical analysis

All quantitative data presented in figures were analyzed using two-tailed *t*-tests in GraphPad Prism 7 software (GraphPad Software, San Diego, CA, USA).

## SUPPLEMENTAL MATERIAL

Supplemental material mbio.01896-25-s0001.docx Supplemental figures and tables.

ASM does not own the copyrights to Supplemental Material that may be linked to, or accessed through, an article. The authors have granted ASM a non-exclusive, world-wide license to publish the Supplemental Material files. Please contact the corresponding author directly for reuse.
