# Supplementary material for: Unculturable bacteria exploit a secretory protein to antagonize insect melanization for persistent infection
Source: mBio. 2025 Aug 29;16(10):e01896-25. doi: 10.1128/mbio.01896-25 (PMC12506112; doi:10.1128/mbio.01896-25)
Supplement: Supplemental material — Supplemental figures and tables. [file mbio.01896-25-s0001.docx]

**Supplemental material**

**
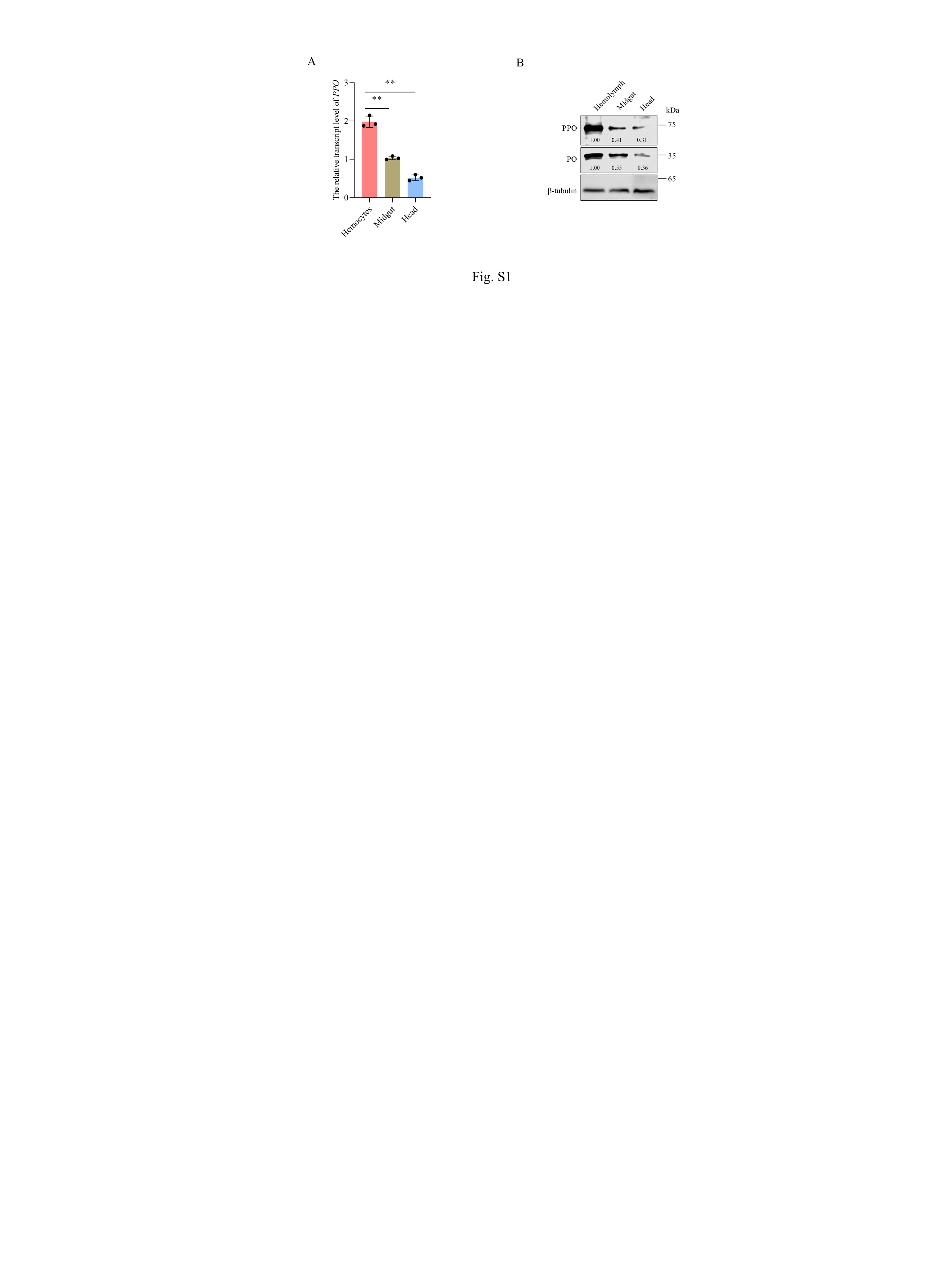
**

**Figure S1 Relative transcript and protein accumulation levels of PPO in various insect organs were assessed using RT-qPCR** (A) **and western blot** (B) **assays.** Data in (A) represent means (± SD) of three replicates, with each replicate containing 30 different organs (two-tailed *t*-test). **, *P*<0.01. The relative intensities of the protein bands were quantified using ImageJ. Insect β-tubulin was used as the loading control. Data represent three replicates, with each replicate containing 30 different organs.


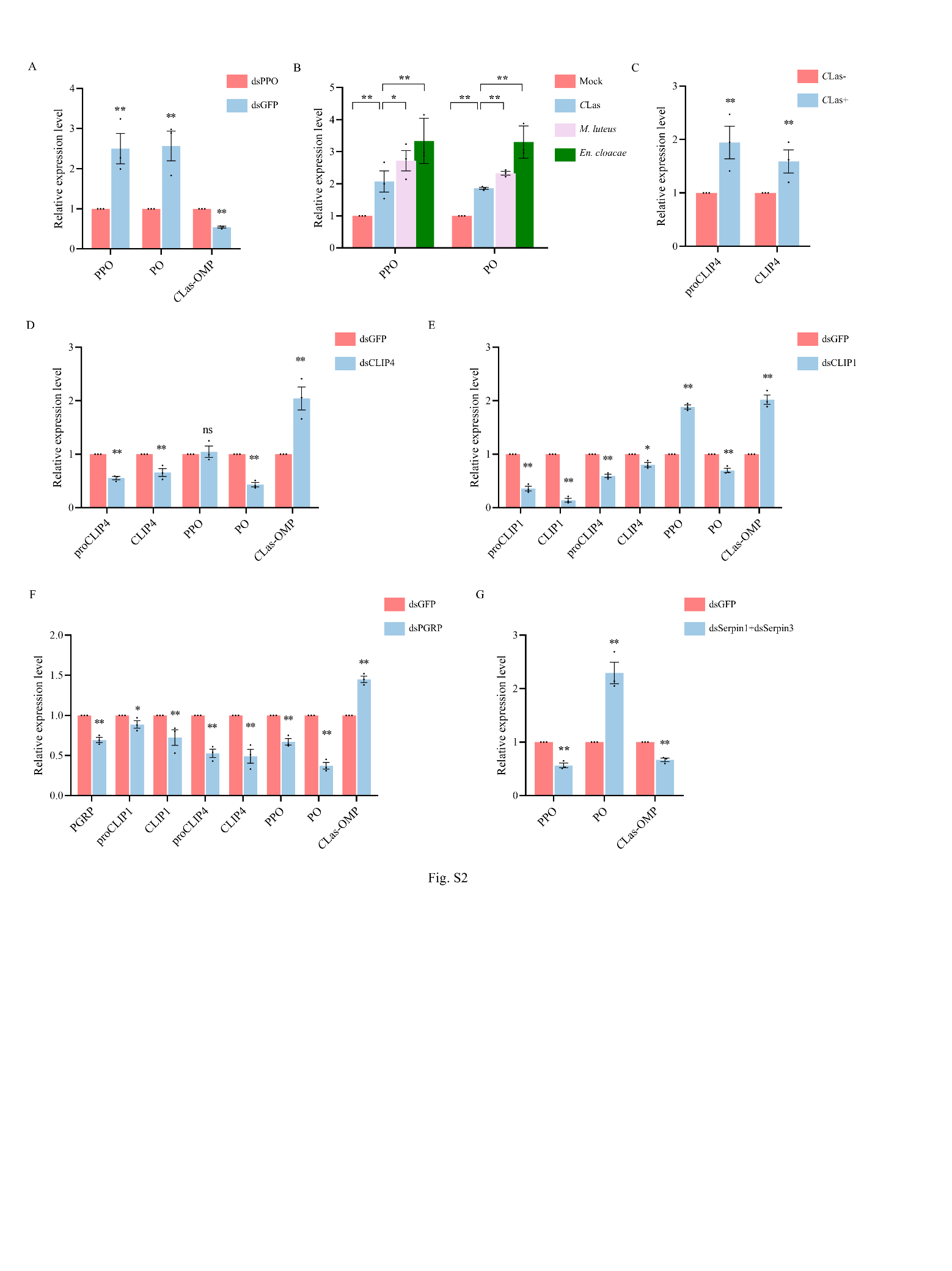


**Figure S2 Quantifications of western blots with ImageJ.** Relatively intensity of protein bands in western blot of Figure 1J (A), 1N (B), 2G (C), 2I (D), 3K (E), 3F (F) and 3N (G).


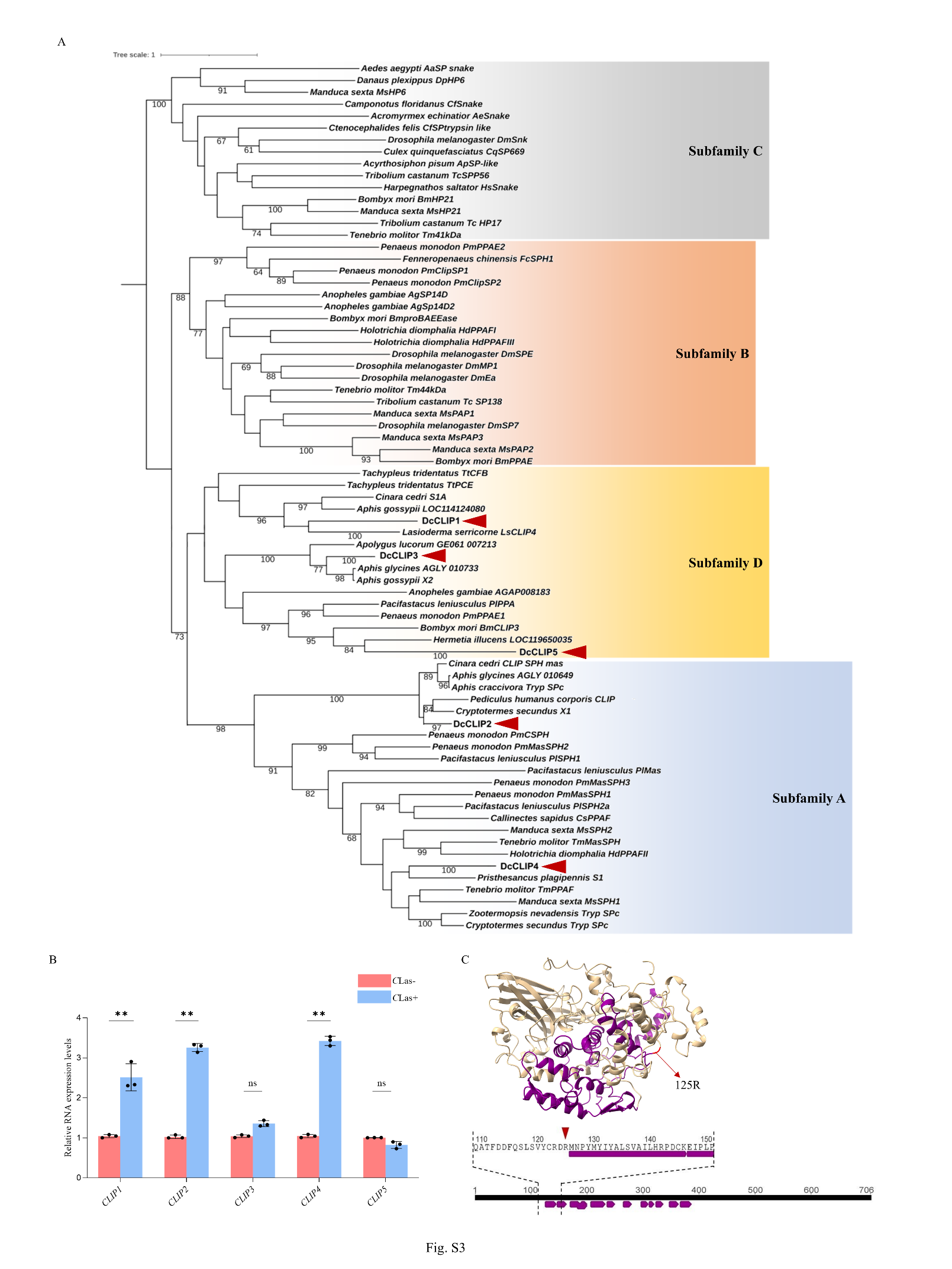


**Figure S3 *C*Las infection induces CLIPs expression in *D. citri*.** (A) Phylogenetic relationships of five CLIPs from *D. citri* with counterparts in various insect species were analyzed using available amino acid sequences. Maximum likelihood phylogenetic trees were reconstructed, with bootstrap values indicated above each node. Arrows indicate the CLIPs from *D. citri*. (B) The relative transcript levels of four different CLIPs were tested by RT-qPCR assays. Data represent means (± SD) of three replicates, with each replicate containing 30 insects (two-tailed *t*-test). ns, not significant; **, *P*<0.01. (C) Predicted structure of PPO using AlphaFold3. The red line and arrow indicate the cleavage site. Purple regions indicate the peptides detected by LC-MS/MS.


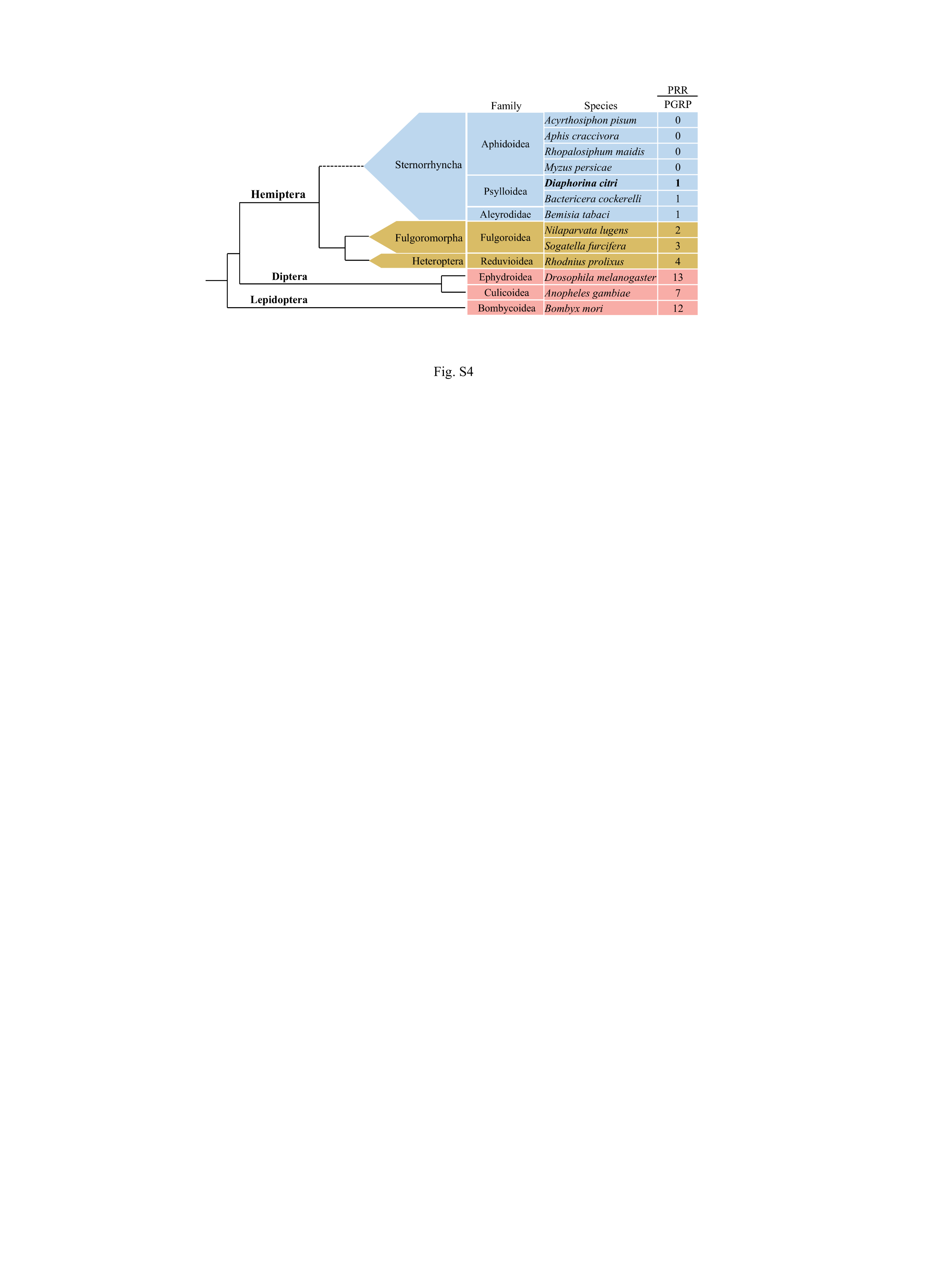


**Figure S4 Bioinformatics analysis of the number of PGRPs across Hemiptera and three representative model insects.**

**
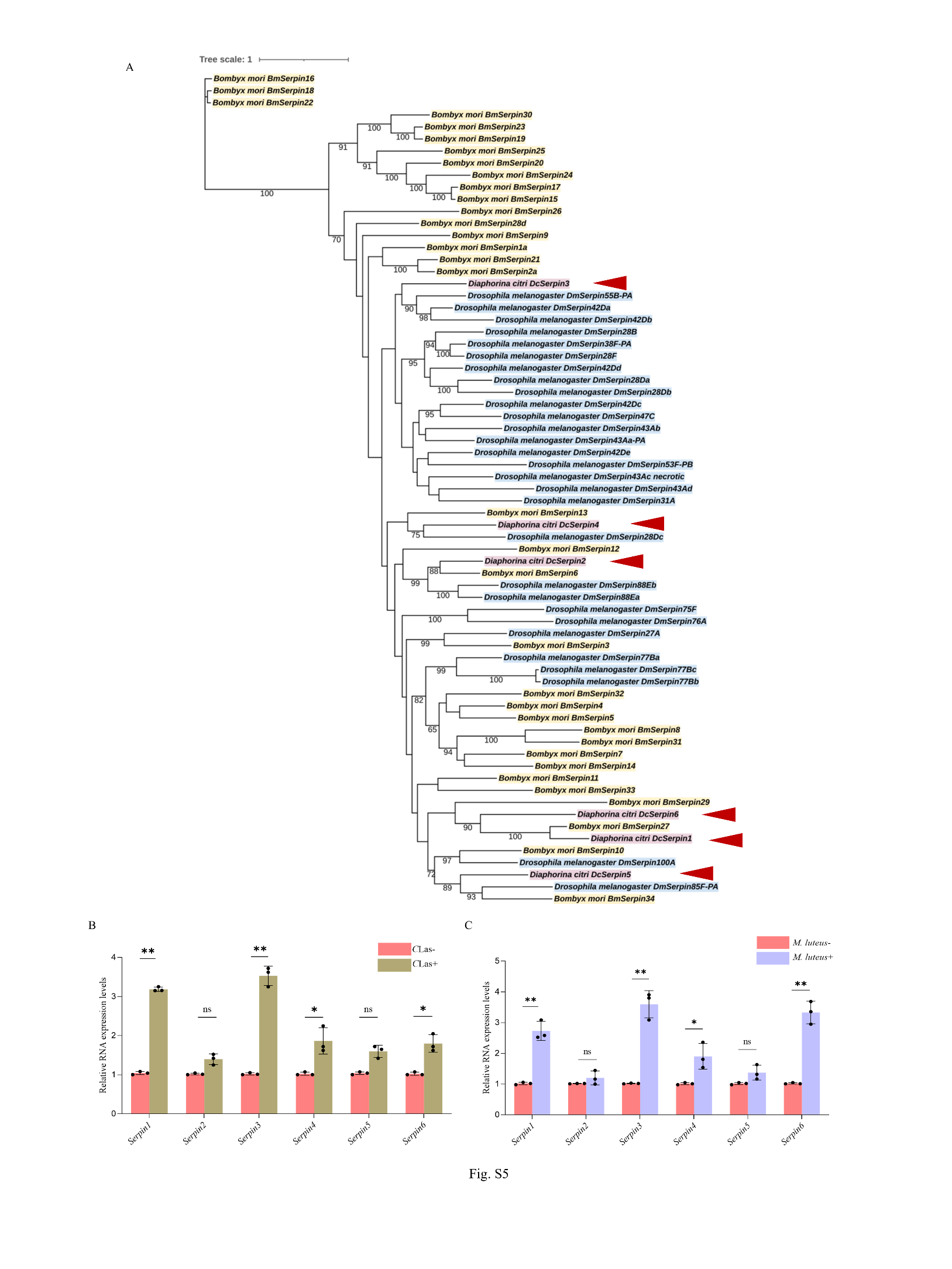
**

**Figure S5 *C*Las infection activates the expression of serpins in *D. citri*.** (A) Phylogenetic relationships of serpins from *D. citri* with those from two representative model insects, *B. mori* and *D. melanogaster*. The available amino acid sequences of *D. citri* serpins and their counterparts were used to reconstruct Maximum likelihood phylogenetic trees, with bootstrap values provided above each node. (B, C) Relative transcript levels of six different serpins in *D. citri* were tested by RT-qPCR assays. Data represent means (± SD) of three replicates, with each replicate containing 30 insects (two-tailed *t*-test). ns, not significant; *, *P*<0.05; **, *P* <0.01.


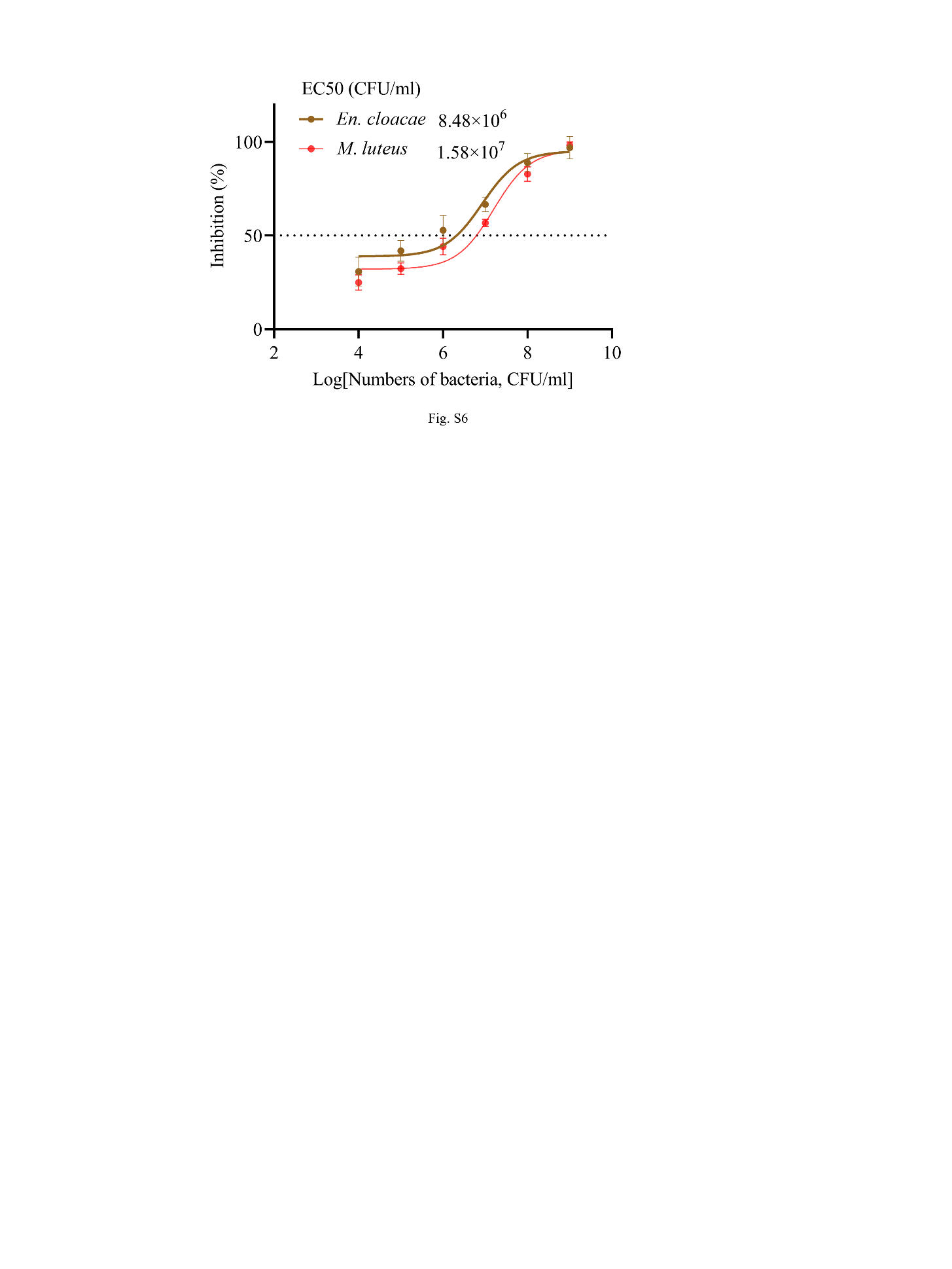


**Figure S6 EC50 of *En. cloacae* and *M. luteus* on *D. citri.***

**Table S1. Primers in this study.**

| Primers | Sequence (5'-3') |
| --- | --- |
| HisDcCLIP1-F | atgggtcgcggatccgaattcGCCCTGTTTCGACTAAGAGAGG |
| HisDcCLIP1-R | ctcgagtgcggccgcaagcttCAGCACACAGAATGTTCTCATTGA |
| HisDcCLIP2-F | atgggtcgcggatccgaattc GATGACAGCTGTTGCATTACCAA |
| HisDcCLIP2-R | ctcgagtgcggccgcaagctt ACAGCTGTGGTTGTAGCCACAG |
| HisDcCLIP3-F | atgggtcgcggatccgaattc ATGGCGGTAAGGAGAGCACA |
| HisDcCLIP3-R | ctcgagtgcggccgcaagctt TCACACCATATTCTGTTCGATCCA |
| HisPPOTB-1-F | atgggtcgcggatccgaattcATGTCCAAGTTCAACCCTACAGATAA |
| HisPPOTB-1-R | ctcgagtgcggccgcaagcttGTTCATGGCATCCCGACAGT |
| HisPPOTB-2-F | atgggtcgcggatccgaattcCGGGATGCCATGAACCCC |
| HisPPOTB-2-R | ctcgagtgcggccgcaagcttTCATTGACGGGCAGGGTTT |
| AD-DcCLIP4-F | gtaccagattacgctcatatgGCAAGATCCCAGAAAAGCTCTG |
| AD-DcCLIP4-R | cagctcgagctcgatggatccTTGGCGGTCTGCTTTCCA |
| BD-PPO-F/BD-PPOM-F | tcagaggaggacctgcatatgATGTCCAAGTTCAACCCTACAGATAA |
| BD-PPO-R | ccgctgcaggtcgacggatccTCATTGACGGGCAGGGTTT |
| BD-PPON-F | tcagaggaggacctgcatatg TTCTCGGTGCCGGCCGAT |
| BD-PPON-R | ccgctgcaggtcgacggatcc CTTGCAGTCAGGCCGATGG |
| BD-PPOC-F | tcagaggaggacctgcatatg CCATACCCGGTGCCTCAAC |
| BD-PPOC-R | ccgctgcaggtcgacggatccGATTTTACAATCCAGGATAGCCATG |
| BD-PPOM-R | ccgctgcaggtcgacggatccACGATCCCGACAGTACACGC |
| HisPPON-F | atgggtcgcggatccgaattc TTCTCGGTGCCGGCCGAT |
| HisPPON-R | ctcgagtgcggccgcaagctt CTTGCAGTCAGGCCGATGG |
| dsDcCLIP4-F | TAATACGACTCACTATAGGGCGAGCAAGATCCCAGAAAAGCTCTG |
| dsDcCLIP4-R | TAATACGACTCACTATAGGGCGATTGGCGGTCTGCTTTCCA |
| BD-DcCLIP1-F | tcagaggaggacctgcatatgGCCCTGTTTCGACTAAGAGAGG |
| BD-DcCLIP1-R | ccgctgcaggtcgacggatccCAGCACACAGAATGTTCTCATTGA |
| GST-DcCLIP4-F | tccgcgtggatccccgaattccGCAAGATCCCAGAAAAGCTCTG |
| GST-DcCLIP4-R | gtcacgatgcggccgctcgagTTGGCGGTCTGCTTTCCA |
| ds-DcCLIP1-F | cccaccatcgggcgcggatccGCCCTGTTTCGACTAAGAGAGG |
| ds-DcCLIP1-R | ctagtacttctcgacaagcttTCACAGATCCTCTTCAGAGATGAGTTTCTGCTCCAGCACACAGAATGTTCTCATTGA |
| ds-PGRP-F | cccaccatcgggcgcggatccGATATAGTCCCGCGGGCTTC |
| ds-PGRP-R | ctagtacttctcgacaagcttTCACAGATCCTCTTCAGAGATGAGTTTCTGCTCGAGTTGACTGTGAGTGAGCAGCTT |
| His-PGRP-F | atgggtcgcggatccgaattcGATATAGTCCCGCGGGCTTC |
| His-PGRP-R | ctcgagtgcggccgcaagcttGAGTTGACTGTGAGTGAGCAGCTT |
| qCLIP1-F | TGATAGGCTGGACCATGGGA |
| qCLIP1-R | GGAACGTGATCTGCGGTGTA |
| qCLIP2-F | CCTCACACATGAAAACTACACTGG |
| qCLIP2-R | AGACCGTCCATCCTCTGTCA |
| qCLIP3-F | TCCAACCAATAACGGACAAC |
| qCLIP3-R | CCAGTAGCCATCTTTCTTCATC |
| qCLIP4-F | GCAGTTTGCCAAAGTTGTC |
| qCLIP4-R | GTCTGCTTTCCATTCCGTC |
| qCLIP5-F | ACCTCGTCTCCTCAAAGTC |
| qCLIP5-R | GTCACTATCTTCACCTTGCTC |
| qSerpin1-F | TGTTGAGAAGTCCACCAGCC |
| qSerpin1-R | CCCTTGTTGGCGTTGTTGAG |
| qSerpin2-F | AGCATATCAGACACAGGCCG |
| qSerpin2-R | GAGGCGTCCAGACTATCAGC |
| qSerpin3-F | CGGTCCGTCATGACCAAGAA |
| qSerpin3-R | CTCGTCCTTAGTTTGGCCGT |
| qSerpin4-F | CTCAACTACGCTACGTCCCC |
| qSerpin4-R | TGGACAGATCAGCTTTGGGC |
| qSerpin5-F | ATCTCGCCATGATGTGGGAC |
| qSerpin5-R | CCGGCACCGATTTTACCTCA |
| qSerpin6-F | CACGTGACGTCCATTCGAGA |
| qSerpin6-R | GAGAGGTTGGGGGTGTTAGC |
| qPPO-F | GCCTATGCCGGAGGCTTATT |
| qPPO-R | GAGGCGTTGACACACTACCA |
| qCLas-16sRNA-F | GGATAACGCATGGAAACGTGTGCT |
| qCLas-16sRNA-R | AATCCAACGCAGGCTCATCTCTCT |
| qactin-F | CCCTGGACTTTGAACAGGAA |
| qactin-R | CTCGTGGATACCGCAAGATT |
| 28aPPON-F | atgggtcgcggatccgaattcCCATACCCGGTGCCTCAAC |
| 28aPPON-R | ctcgagtgcggccgcaagcttGATTTTACAATCCAGGATAGCCATG |
| 28aPPOC-F | atgggtcgcggatccgaattcATGAACCCCTACATGTACATCTATGC |
| 28aPPOC-R | ctcgagtgcggccgcaagcttTCGATTATCAGGATCGTGGCA |
| 28aPPOM-F/HisPPO-F | atgggtcgcggatccgaattcATGTCCAAGTTCAACCCTACAGATAA |
| 28aPPOM-R | ctcgagtgcggccgcaagcttACGATCCCGACAGTACACGC |
| dsPPO-F | TAATACGACTCACTATAGGGCGATGGTAGTGTGTCAACGCCTC |
| dsPPO-R | TAATACGACTCACTATAGGGCGATCTATCGAAGGGGAAGCCCA |
| AD-SDE3230-F | gtaccagattacgctcatatg ATGAACTTCAGAATAGCGATGTTAATATC |
| AD-SDE3230-R | cagctcgagctcgatggatcc TCAGGGACATAAACCCTTTGACG |
| GSTSDE3230-F | tccgcgtggatccccgaattcc ATGAACTTCAGAATAGCGATGTTAATATC |
| GSTSDE3230-R | gtcacgatgcggccgctcgag TCAGGGACATAAACCCTTTGACG |
| HisSDE3230-F | atgggtcgcggatccgaattc ATGAACTTCAGAATAGCGATGTTAATATC |
| HisSDE3230-R | ctcgagtgcggccgcaagctt TCAGGGACATAAACCCTTTGACG |
| HisPPO-R | ctcgagtgcggccgcaagcttTCATTGACGGGCAGGGTTT |
| pFastBaC1-F | cccaccatcgggcgcggatcc |
| pFastBaC1-His-R | ctagtacttctcgacaagcttTCAATGGTGATGGTGATGATG |
| pFastBaC1-Flag-R | ctagtacttctcgacaagcttTCACTTGTCATCGTCGTCCTTGTAGTC |
| pFastBaC1-Myc-R | tagtacttctcgacaagcttTCACAGATCCTCTTCAGAGATGAGTTTCTGCTC |

**Table S2. NCBI accession numbers of genes in this study.**

| Gene | NCBI accession number |
| --- | --- |
| *DcCLIP4* | XP_008470938 |
| *DcCLIP2* | XP_008488274 |
| *DcCLIP5* | XP_008488275 |
| *DcCLIP1* | XP_017303373 |
| *DcCLIP3* | XP_026676042 |
| *PGRP* | XM_026822519 |
| *PPO* | KAI5702728 |
| *Serpin1* | XP_008487511 |
| *Serpin2* | XP_026678781 |
| *Serpin3* | XP_026686116 |
| *Serpin4* | XP_026677956 |
| *Serpin5* | XP_008472813 |
| *Serpin6* | XP_017300323 |

**Table S3. Quantifications of the Western blot analysis with ImageJ in this study.**

| Figure number | Antibody | Lane (left to right in figure) | Band densitometry |
| --- | --- | --- | --- |
| 1-C | PPO | 1 | 63830.80 |
|  |  | 2 | 56304.73 |
|  | PO | 1 | 45041.24 |
|  |  | 2 | 4906.61 |
|  | *C*Las OMP | 1 | 55640.84 |
|  | β-tubulin | 1 | 55101.89 |
|  |  | 2 | 54640.92 |
| 1-J | PPO | 1 | 18502.33 |
|  |  | 2 | 36888.62 |
|  | PO | 1 | 19016.72 |
|  |  | 2 | 34711.92 |
|  | *C*Las OMP | 1 | 30123.40 |
|  |  | 2 | 16815.94 |
|  | β-tubulin | 1 | 45610.68 |
|  |  | 2 | 42241.73 |
| 1-N | PPO | 1 | 10987.02 |
|  |  | 2 | 29348.77 |
|  |  | 3 | 35577.97 |
|  |  | 4 | 45561.79 |
|  | PO | 1 | 12532.15 |
|  |  | 2 | 23473.02 |
|  |  | 3 | 27842.20 |
|  |  | 4 | 38333.85 |
|  | β-tubulin | 1 | 41512.21 |
|  |  | 2 | 27779.07 |
|  |  | 3 | 24870.85 |
|  |  | 4 | 23532.51 |
| 2-A | PPO | 1 | 44862.69 |
|  |  | 2 | 38767.60 |
|  |  | 3 | 22757.48 |
|  | PO | 1 | 48919.84 |
|  | β-tubulin | 1 | 39337.46 |
|  |  | 2 | 42617.62 |
|  |  | 3 | 45207.79 |
| 2-D | PPO/mPPO | 1 | 32283.40 |
|  |  | 2 | 49568.87 |
|  |  | 3 | 43262.11 |
|  | PO | 1 | 29427.21 |
|  |  | 2 | 7665.25 |
|  |  | 3 | 3705.79 |
|  | β-tubulin | 1 | 48791.22 |
|  |  | 2 | 47067.40 |
|  |  | 3 | 35992.04 |
| 2-G | proCLIP4 | 1 | 17087.26 |
|  |  | 2 | 42180.53 |
|  | CLIP4 | 1 | 25003.69 |
|  |  | 2 | 48656.66 |
|  | β-tubulin | 1 | 49157.21 |
|  |  | 2 | 58492.71 |
| 2-I | proCLIP4 | 1 | 33175.21 |
|  |  | 2 | 19563.05 |
|  | CLIP4 | 1 | 33411.50 |
|  |  | 2 | 17708.97 |
|  | PPO | 1 | 32233.77 |
|  |  | 2 | 40272.12 |
|  | PO | 1 | 36363.32 |
|  |  | 2 | 13283.85 |
|  | *C*Las OMP | 1 | 15642.24 |
|  |  | 2 | 37669.71 |
|  | β-tubulin | 1 | 37634.14 |
|  |  | 2 | 40207.19 |
| 3-C | proCLIP1 | 1 | 31758.55 |
|  |  | 2 | 55921.92 |
|  | CLIP1 | 1 | 11090.12 |
|  |  | 2 | 49078.06 |
|  | β-tubulin | 1 | 44881.55 |
|  |  | 2 | 46459.70 |
| 3-D | proCLIP4 | 1 | 44778.84 |
|  |  | 2 | 25799.87 |
|  |  | 3 | 31207.99 |
|  | CLIP4 | 1 | 44288.63 |
|  |  | 2 | 46318.38 |
|  |  | 3 | 37705.76 |
| 3-F | proCLIP1 | 1 | 38925.42 |
|  |  | 2 | 11467.41 |
|  | CLIP1 | 1 | 42715.52 |
|  |  | 2 | 4032.25 |
|  | proCLIP4 | 1 | 46683.71 |
|  |  | 2 | 24990.89 |
|  | CLIP4 | 1 | 29579.64 |
|  |  | 2 | 26142.59 |
|  | PPO | 1 | 24616.77 |
|  |  | 2 | 44697.77 |
|  | PO | 1 | 42753.63 |
|  |  | 2 | 27870.56 |
|  | *C*Las OMP | 1 | 22581.19 |
|  |  | 2 | 49376.31 |
|  | β-tubulin | 1 | 49145.35 |
|  |  | 2 | 38969.48 |
| 3-I | PGRP | 1 | 34974.72 |
|  |  | 2 | 43365.87 |
|  | β-tubulin | 1 | 44498.36 |
|  |  | 2 | 41670.58 |
| 3-K | PGRP | 1 | 36967.29 |
|  |  | 2 | 25674.94 |
|  | proCLIP1 | 1 | 30401.43 |
|  |  | 2 | 26740.34 |
|  | CLIP1 | 1 | 22698.84 |
|  |  | 2 | 11989.58 |
|  | proCLIP4 | 1 | 34623.77 |
|  |  | 2 | 14720.70 |
|  | CLIP4 | 1 | 33202.81 |
|  |  | 2 | 10951.92 |
|  | PPO | 1 | 35997.48 |
|  |  | 2 | 22389.31 |
|  | PO | 1 | 40017.79 |
|  |  | 2 | 14187.05 |
|  | *C*Las OMP | 1 | 23765.68 |
|  |  | 2 | 32693.74 |
|  | β-tubulin | 1 | 45941.17 |
|  |  | 2 | 41611.19 |
| 3-N | PPO | 1 | 41170.05 |
|  |  | 2 | 21869.74 |
|  | PO | 1 | 18909.77 |
|  |  | 2 | 50904.14 |
|  | CLAS OMP | 1 | 43364.02 |
|  |  | 2 | 30700.43 |
|  | β-tubulin | 1 | 43731.82 |
|  |  | 2 | 48574.74 |
| 4-A | SDE3230 | 1 | 37007.27 |
|  | β-tubulin | 1 | 44451.51 |
|  |  | 2 | 47235.80 |
| 4-Ei | GST-PPO | 1 | 37771.29 |
|  |  | 2 | 38086.90 |
|  |  | 3 | 35399.92 |
|  |  | 4 | 30820.66 |
|  | His-SDE3230 | 1 | 13543.97 |
|  |  | 2 | 22430.95 |
|  |  | 3 | 30397.90 |
|  |  | 4 | 39349.85 |
|  | His-CLIP4 | 1 | 32953.63 |
|  |  | 2 | 33561.73 |
|  |  | 3 | 36018.68 |
|  |  | 4 | 37643.42 |
|  | His-CLIP4 | 1 | 42548.68 |
|  |  | 2 | 32419.63 |
|  |  | 3 | 21677.61 |
|  |  | 4 | 8749.12 |
| 4-E-ii | GST-PPO | 1 | 34897.89 |
|  |  | 2 | 33037.05 |
|  |  | 3 | 37536.39 |
|  |  | 4 | 30718.54 |
|  | His-SDE3230 | 1 | 27703.44 |
|  |  | 2 | 28555.87 |
|  |  | 3 | 33957.65 |
|  |  | 4 | 24125.37 |
|  | His-CLIP4 | 1 | 16243.34 |
|  |  | 2 | 24105.12 |
|  |  | 3 | 25839.10 |
|  |  | 4 | 37907.41 |
|  | His-SDE3230 | 1 | 24565.12 |
|  |  | 2 | 22481.27 |
|  |  | 3 | 23344.44 |
|  |  | 4 | 27214.46 |
| 4-F | PPO | 1 | 35476.16 |
|  |  | 2 | 22659.99 |
|  | PO | 1 | 17362.69 |
|  |  | 2 | 29340.99 |
| 4-G | SDE3230 | 1 | 46790.81 |
|  | PPO | 1 | 31345.05 |
|  |  | 2 | 46220.09 |
|  | PO | 1 | 40696.41 |
|  |  | 2 | 10413.58 |
|  | β-tubulin | 1 | 42224.40 |
|  |  | 2 | 48143.89 |
| 4-I | PPO | 1 | 30449.14 |
|  |  | 2 | 30855.84 |
|  | PO | 1 | 43727.99 |
|  |  | 2 | 18062.12 |
|  | *C*Las OMP | 1 | 13306.07 |
|  |  | 2 | 34433.42 |
|  | β-tubulin | 1 | 47542.40 |
|  |  | 2 | 36117.76 |
| 5-F | PPO | 1 | 18153.82 |
|  |  | 2 | 26482.65 |
|  |  | 3 | 31868.19 |
|  |  | 4 | 37243.12 |
|  | PO | 1 | 18972.70 |
|  |  | 2 | 25079.77 |
|  |  | 3 | 40764.29 |
|  |  | 4 | 35417.48 |
|  | *C*Las OMP | 1 | 34532.80 |
|  |  | 2 | 29764.46 |
|  |  | 3 | 9079.71 |
|  |  | 4 | 15390.46 |
|  | β-tubulin | 1 | 31343.17 |
|  |  | 2 | 23954.07 |
|  |  | 3 | 31293.37 |
|  |  | 4 | 28410.63 |
| 7-B | PPO | 1 | 37520.53 |
|  |  | 2 | 9261.20 |
|  |  | 3 | 4200.10 |
|  | PO | 1 | 40981.60 |
|  |  | 2 | 24857.58 |
|  |  | 3 | 7640.66 |
|  | β-tubulin | 1 | 35236.92 |
|  |  | 2 | 31584.72 |
|  |  | 3 | 49844.84 |
